# DNA Motifs Are Not General Predictors of Recombination in Two *Drosophila* Sister Species

**DOI:** 10.1093/gbe/evz082

**Published:** 2019-04-15

**Authors:** James M Howie, Rupert Mazzucco, Thomas Taus, Viola Nolte, Christian Schlötterer

**Affiliations:** 1 Institut für Populationsgenetik, Vetmeduni Vienna, Austria; 2 Vienna Graduate School of Population Genetics, Vetmeduni Vienna, Austria

**Keywords:** *D. simulans*, genomic correlation, linkage disequilibrium, motif density, motif model, recombination map

## Abstract

Meiotic recombination is crucial for chromosomal segregation and facilitates the spread of beneficial and removal of deleterious mutations. Recombination rates frequently vary along chromosomes and *Drosophila melanogaster* exhibits a remarkable pattern. Recombination rates gradually decrease toward centromeres and telomeres, with a dramatic impact on levels of variation in natural populations. Two close sister species, *Drosophila simulans* and *Drosophila mauritiana* do not only have higher recombination rates but also exhibit a much more homogeneous recombination rate that only drops sharply very close to centromeres and telomeres. Because certain sequence motifs are associated with recombination rate variation in *D. melanogaster*, we tested whether the difference in recombination landscape between *D. melanogaster* and *D. simulans* can be explained by the genomic distribution of recombination rate–associated sequence motifs. We constructed the first high-resolution recombination map for *D. simulans* based on 189 haplotypes from a natural *D. simulans* population and searched for short sequence motifs linked with higher than average recombination in both sister species. We identified five consensus motifs significantly associated with higher than average chromosome-wide recombination rates in at least one species and present in both. Testing fine resolution associations between motif density and recombination, we found strong and positive associations genome-wide over a range of scales in *D. melanogaster*, while the results were equivocal in *D. simulans*. Despite the strong association in *D. melanogaster*, we did not find a decreasing density of these short-repeat motifs toward centromeres and telomeres. We conclude that the density of recombination-associated repeat motifs cannot explain the large-scale recombination landscape in *D. melanogaster*, nor the differences to *D. simulans*. The strong association seen for the sequence motifs in *D. melanogaster* likely reflects their impact influencing local differences in recombination rates along the genome.

## Introduction

Meiotic recombination rate variation impacts on multiple important biological processes in sexual eukaryotes. It is crucial for chromosomal segregation (Roeder 1997; John 2005) but is also itself a powerful factor influencing genome organization and sequence variability (Aquadro et al. 1994; True et al. 1996). Meiotic recombination arises when a double-stranded break (DSB) leads to crossing over between homologous chromatids (Szostak et al. 1983; Schwacha and Kleckner 1995; Bergerat et al. 1997; Keeney et al. 1997; Hughes et al. 2018). Higher rates of recombination break up genetic linkage and can increase the efficacy of natural selection (Haddrill et al. 2007; Charlesworth and Charlesworth 2010) and so affect the evolution of numerous genomic features. The reduction of transposable element density (Charlesworth et al. 1992, 1994; Rizzon et al. 2002; Petrov et al. 2011; Kofler et al. 2012) and the increased levels of DNA polymorphism in regions of high recombination (Begun and Aquadro 1992; Aquadro et al. 1994; Begun et al. 2007; Kulathinal et al. 2008) are probably the clearest examples.

Yet although the eukaryotic meiotic machinery is generally highly conserved (Keeney 2001), rates of recombination have been observed to vary dramatically across species and populations, between individuals, and across sexes (Stapley et al. 2017; Haenel et al. 2018), apparently due to a combination of interacting environmental, epigenetic, and genetic factors (Detlefsen and Roberts 1921; Stern 1926; Neel 1941; Parsons 1958; Stapley et al. 2017). Moreover, the distribution of meiotic recombination rates among and along chromosomes varies markedly across taxa (Lichten and Goldman 1995; Petes 2001; Hey 2004; Choi and Henderson 2015; Hunter, Huang, et al. 2016; Stapley et al. 2017). Large-scale recombination suppression is often observed toward centromeres, the so-called “centromere effect” (Beadle 1932; Szauter 1984; Choulet et al. 2014; Hughes et al. 2018). Depending on the species, either suppression or enhancement of recombination has been observed toward the telomeres (Broman et al. 1998; Myers et al. 2005; Chan et al. 2012; Comeron et al. 2012). Heterochromatin, which is often associated with these regions, tends also to exhibit lower recombination rates than euchromatin (Sturtevant and Beadle 1936; Baker 1958; Roberts 1965; Szauter 1984; Termolino et al. 2016). Yet, in addition to these large-scale features of recombination landscapes, fast-evolving (Jeffreys et al. 2001) finer-scale variation can also be observed (Myers et al. 2005; Comeron et al. 2012).

It has been proposed that short sequence motifs are a key factor shaping the recombination landscape. For example, in humans a 13-mer, CCNCCNTNNCCNC motif is targeted by the PRDM9 protein (Myers et al. 2010; Grey et al. 2011; Billings et al. 2013), via its zinc-finger array (Baudat et al. 2010; Parvanov et al. 2010), where it promotes histone methylation and meiotic crossover, reorganizing the nucleosome around it and driving DSB formation (Mihola et al. 2009; Brick et al. 2012; Baker et al. 2014; Pratto et al. 2014). These highly localized recombination events in 500–2,000-bp sections of chromosome have been called recombination “hotspots” (Lam and Keeney 2015). They are observed in a multitude of species including yeast, mice, humans among many others (Lam and Keeney 2015).

Hotspots are, however, no universal feature of recombination landscapes and are not observed in a range of species groups including *Caenorhabditis elegans* and *Drosophila* (Aquadro et al. 2001; Nachman 2002; Hey 2004; Chan et al. 2012; Manzano-Winkler et al. 2013; Heil et al. 2015; Miller et al. 2016). *Drosophila* spp. exhibit a large heterogeneity in recombination across their chromosomes, as demonstrated in *D. persimilis* (Stevison and Noor 2010), *D. pseudoobscura* (Cirulli et al. 2007; Kulathinal et al. 2008), and *D. melanogaster* (Singh et al. 2009; Comeron et al. 2012; Adrian et al. 2016). Still, *D. melanogaster* exhibits only a handful of mild “hotspots” relative to the ∼30,000, often very strong hotspots observed in humans (International HapMap Consortium 2007). Instead, the *D. melanogaster* recombination landscape is characterized by recombination “peaks” and “valleys” on a 5–500-kb scale (Singh et al. 2009; Chan et al. 2012; Comeron et al. 2012; Adrian et al. 2016), with which short “recombination motifs” are associated; as has also been seen in *D. pseudoobscura*, *D. persimilis*, and other *Drosophila* species (Cirulli et al. 2007; Kulathinal et al. 2008; Singh et al. 2009; Stevison and Noor 2010; Chan et al. 2012; Comeron et al. 2012; Heil and Noor 2012; Miller et al. 2012; Singh et al. 2013; Adrian et al. 2016). These repeat motifs, which often reside in transcription-associated euchromatic regions (Petes 2001; Comeron et al. 2012), are thought to increase the accessibility of DNA chromatin to double-stranded cleavage (Comeron et al. 2012) and destabilize DNA sequences, potentially in a stress, environmental or epigenetically dependent manner (Stern 1926; Neel 1941; Redfield 1966; Petes 2001; Hunter, Robinson, et al. 2016; Kohl and Singh 2018).

*Drosophila melanogaster*, *D. simulans*, and *D. mauritiana* are sister species which are ecologically and karyotypically similar (Lemeunier and Ashburner 1976; True et al. 1996) but differ dramatically in their recombination landscapes. Although *D. melanogaster* exhibits a characteristic gradual decrease in recombination rate toward centromeres and to a lesser extent also telomeres, the recombination landscape in *D. simulans* and *D. mauritiana* is much flatter with a rather constant recombination rate almost to the end of the chromosome arm, where it drops very quickly (True et al. 1996). Furthermore, these two species also have a higher recombination rate than *D. melanogaster* (True et al. 1996), which has been attributed, in *D. mauritiana*, to the MEI-218 protein which has highly diverged between *D. melanogaster* and *D. mauritiana*, and promotes recombination to a greater extent in the latter (Brand et al. 2018).

Here, to test the hypothesis that differences in genome-wide motif distributions can explain the observed differences in recombination rates (Adrian et al. 2016), we take a multistep approach. First, we produce a high-resolution recombination map for *D. simulans* from 189 haplotypes. Next, we run a motif discovery in *D. melanogaster* and *D. simulans* and construct a consensus motif set for repeat motifs associated with recombination at the broadest scale, when each chromosome is dived into higher and lower than median recombination regions (each ∼12,000 kb). We confirm the clear differences in recombination landscapes between the two species but find a similar set and distribution of recombination-associated motifs in each. Analyzing motif-recombination associations at a range of finer scales (1, 5, 25, 101, 501, and 2,501 kb), our results suggest that recombination-associated motifs cannot explain the large-scale differences in recombination landscapes between the two species but may have a significant impact on recombination at a local scale, in particular in *D. melanogaster*.

## Materials and Methods

### Recombination Map

#### Recombination Map Production

A total of 202 isofemale lines were established from a natural *D. simulans* population in Tallahassee, FL, USA, in 2010 (Barghi et al. 2017). From each of the 189 lines that were still alive in 2016, an individual male was selected and crossed with a virgin “reference” female from the M252 strain that was used to produce the *D. simulans* reference genome (Palmieri et al. 2015). DNA from individual F1 female offspring from each cross was then extracted using NEBNext Ultra II DNA Library Prep Kits (E7645, New England Biolabs). One hundred eighty-nine paired-end Illumina libraries were generated from single F1 females, with an average insert size of 260 bp, and sequenced on an Illumina HiSeq XTEN to obtain an average sequence coverage of 30×. Single-nucleotide polymorphisms (SNPs) were called with FreeBayes (v1.1.0-46-g8d2b3a0, Garrison and Marth 2012) relative to the *D. simulans* reference genome (Palmieri et al. 2015), requiring a minimum sequencing coverage of 10× and a variant quality of at least 50. All single-nucleotide polymorphisms that were polymorphic in the M252 reference strain were masked. Based on line-specific haplotype information, the genome-wide recombination map was estimated with LDJump (v0.1.4, Hermann et al. 2019), specifying a segment size of 1 kb, with an *α* = 0.05 and an Θ= 0.04. We disabled LDJump’s segmentation analysis and worked with raw recombination rate estimates. Recombination rates were converted from *ρ* (rho) to units of cM/Mb by normalizing them so as to have a genetic map length between a set of marker genes equivalent to that which has been previously reported (True et al. 1996). The recombination map therefore represents an average of the recombination rate across both males and females in a population, normalized to females.

The resultant *D. simulans* recombination map was used in parallel with the *D. melanogaster* recombination map produced by Comeron et al. (2012), downloaded from the *D. melanogaster* Recombination Rate Calculator (Fiston-Lavier et al. 2010, last accessed April 19, 2019 ).

#### Recombination Map Scaling

As the raw recombination map output by LDJump is noisy, we smoothed each recombination map at several scales. In *D. melanogaster*, the raw map (Comeron et al. 2012) contained information on recombination rate at a 100-kb resolution, in *D. simulans* raw information was generated at a 1-kb scale. For smoothing, we used a moving median approach, using window sizes of 5, 25, 101, 501, and 2,501 kb for *D. simulans*, and of 101, 501, and 2,501 kb for *D. melanogaster*, respectively. It is important to note that identical smoothing was performed in both species, though for a larger number of finer resolutions in *D. simulans* due to the higher resolution of the available data, such that species differences cannot arise due to different smoothing procedures. Advantages of the moving median as a smoothing method include low sensitivity to outliers, and a direct relationship to underling data, in the sense that only values present in the raw data set can be present in the smoothed set if the median is taken based on an odd number of input values, which in our case it always was. The moving median is preferred to a moving mean due to a reduced sensitivity to outliers. Because this approach is computationally expensive and prone to deleting map features when there are long runs of identical values, we investigated, as an alternative approach, smoothing via LOESS local regression (Cleveland et al. 1992), which produces qualitatively equivalent results (supplementary fig. S2, Supplementary Material online). The smoothing scales chosen reflect those in Adrian et al. (2016), as relevant to potential motif explanatory power. The “correct” scale on which motifs may function is a priori unclear. A key point of our study is comparing results across several scales.

### DNA Motif Identification

#### Motif Discovery

For each species, we ran a genome-wide motif discovery using MEME (Bailey and Elkan 1994), from the MEME suite of motif-based sequence analysis tools (Bailey et al. 2009, version 5.0.1pl, accessible at http://meme-suite.org last accessed April 19, 2019; Bailey et al. 2015), a software designed to detect DNA sequence motifs in genetic data. After dividing each of the five large chromosomes (X, 2L, 2R, 3L, and 3R) into high- and low-recombining regions based on the chromosome median recombination rate, we used this software in the “differential enrichment” mode to detect motifs enriched in high-recombining areas of the genome. For *D. melanogaster*, we ran MEME on the release 5 reference genome (v*.* 5.36), for concordance with our recombination information from (Comeron et al. 2012). For *D. simulans*, we used the M252 Madagascar reference genome (Palmieri et al. 2015), to align with our recombination map. Motif discovery searches were run with species-specific Markov Background Models, simple matrices of background base frequencies obtained using the MEME *fasta-get-model* command, for each reference genome in turn. The full procedure was repeated with all smoothed maps (Recombination Map Production section). For completeness, a raw 1-kb window motif discovery run was also conducted for *D. simulans*. A similar search for motifs associated with lower recombination areas returned no results.

#### Motif Consensus Set

MEME motif discovery runs returned a set of 5, 4, and 3 motifs in *D. melanogaster* and 1, 2, 4, 1, 1, and 1 significant motifs in *D. simulans*, at the 101, 501, and 2,501, and 1, 5, 25, 101, 501, and 2,501-kb scales, respectively (supplementary S3, Supplementary Material online, *E *≤* *0.01). That is, at least some motifs were recovered at all scales in both species, though fewer size scales were tested in *D. melanogaster* due to its coarser map resolution (see supplementary S3, Supplementary Material online, for a full description of all motifs recovered at all scales). It was noticed that, while individually distinct, numerous motifs contained similar core patterns while varying, for example, only in repeat number. As such, we constructed a set of five consensus motifs that captured the core variation in all motifs significantly associated with increased recombination, across both species, and over all scales. This core set of motifs C1–5 was determined via a two-step method. First, we contrasted the motifs across each of our recombination map smoothing scales in both species, retaining only motifs that occurred in at least one scale with a minimum significance of *E *≤* *0.01 in at least one species. Motifs were then simplified by allowing only the most likely base at each position, and motif lengths were fixed as the longest sequence length that could be represented in both species (as lengths were by tendency longer in *D. melanogaster*). This resulted in the following set of consensus motifs: C1 = [A]_11_; C2 = [GCA]_4_; C3 = [CA]_6_; C4 = [TA]_5_; C5 = [G]_8_, C1–3 and C5 significant in both species, and C4 in *D. melanogaster* only (supplementary S3, Supplementary Material online). We note that *D. melanogaster* made the dominant contribution to the consensus motifs, as the motifs in *D. simulans* were less significant than those observed in *D. melanogaster* (supplementary S3, Supplementary Material online), and that the number of consensus motifs was informed by the data, and not decided a priori. As our consensus motifs turned out to be simplified versions of the most predictive motifs that were identified by Adrian et al. (2016), a subset of those sequence motifs identified in an earlier article by Comeron et al. (2012), we quantitatively tested and confirmed this similarity using the MEME Suite tool TomTom (Gupta et al. 2007), under default parameters (supplementary S4, Supplementary Material online).

### Genome-Wide Motif Densities

#### Motif Locations

We converted the five consensus motifs into letter-probability matrices, to be used as input to Find Individual Motif Occurrances or FIMO, a MEME Suite tool designed to find genome-wide motif occurrences (Grant et al. 2011). Matrices were compiled in a hard, and a softer, version; with the expected base given a probability of 1 and unexpected bases probabilities of 0, or the expected base a probability of 0.97, and unexpected bases each a probability of 0.01. FIMO was then run for each species, taking the reference sequences and Markov Background Models as noted in the Motif Discovery section, and using parameter *max-stored-scores* = 50,000,000, and all others at default. Results of the hard and soft motif probability runs were qualitatively identical, so hard coded motif probabilities were used for follow-up analysis (soft runs are not here reported).

#### Motif Densities

FIMO output provides, per motif, the genomic locations (chromosome, start and stop position) at which a motif was found, as well as a *P* value and a *q*-score (Benjamini and Hochberg 1995) per record, which show how well the motif was matched to the underlying reference sequence, respectively before and after correction for multiple testing (Benjamini and Hochberg 1995). To obtain genome-wide motif densities in each species, we calculated for each motif the sum of 1 – *q*, across a sliding window of 1 kb, where *q* refers to the per record *q*-score, such that per window motif densities are discounted in relation to the quality of the motif match, with higher quality matches counting more. A total, genome-wide count (of 1 – *q*) of each motif was also obtained from the raw FIMO output. These motif densities were obtained for windows of 1, 5, 25, 101, 501, and 2,501-kb in *D. simulans* and at 101, 501, and 2,501 kb in *D. melanogaster*. We note that the scales at which the motifs were identified are far larger than the windows used for these motif density calculations.

### Motif–Recombination Correlations and Models

#### Motif Density–Recombination Rate Correlations

To investigate the relationship between recombination rates and genome-wide abundances of individual motifs, we calculated the correlations between motif densities, binned at 1 kb, and corresponding recombination rates (cM/Mb), per motif, for *D. simulans* and *D. melanogaster*, respectively. As there was no clear a priori expectation for the genomic scale at which motifs would have most impact on recombination, the analysis was repeated for all three and five smoothing scales noted in the Recombination Map Scaling section for *D. melanogaster* and *D. simulans*, respectively (and was also repeated on the raw 1-kb scale in for *D. simulans*, not shown). The analysis was conducted using an identical procedure in each species, though at fewer scales in *D. melanogaster* due to limited resolution of the Comeron et al. (2012) recombination map. Spearman’s rho, *ρ*, was used as a nonparametric estimator of the correlation between the test variables, and both the direction and significance of all correlations were extracted. To investigate the overall predictive power of motif densities, irrespective of chromosomal background, the analysis was repeated on the total genomic data, pooling across all of the five major chromosomes, with the analysis repeated per motif and species.

Finally, to test for explicit directional effects of each consensus motif on recombination, a linear regression model was fitted, per motif, species, scale, and chromosome, for the effect of motif density on local recombination rate, and repeated for the genome average.

**Fig. 1.**
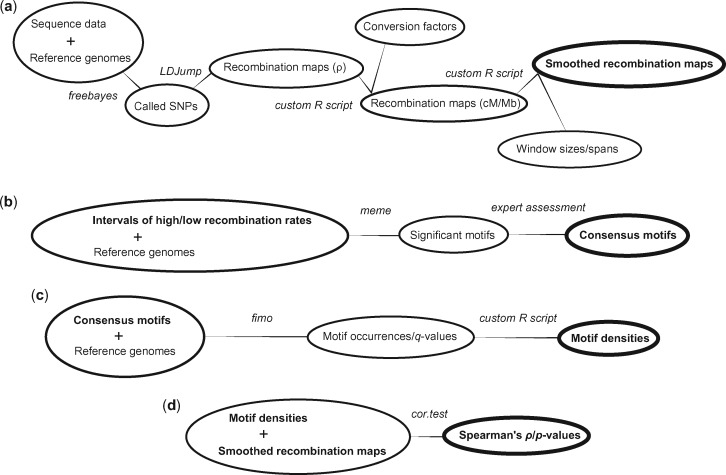
—A schematic representation of the bioinformatic pipeline used. Ovals represent physical data sets, lines represent tools used to derive them; see the Materials and Methods for details.

A schematic representation of this analytic pipeline is presented in figure 1. All statistical analyses were run in R, version 1.1383 (R Core Development Team 2018), using in house scripts (fully available under supplementary S5, Supplementary Material online).

## Results

### Recombination Rates in *D. simulans* Are More Uniform across Chromosomes, than in *D. melanogaster*

**Fig. 2.**
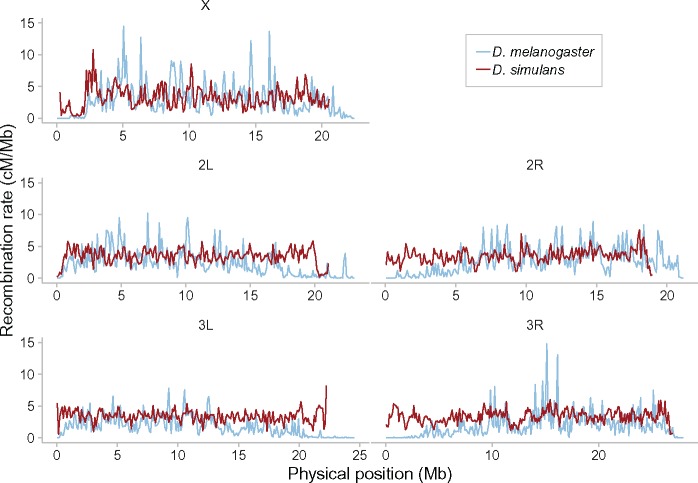
—Recombination rates in *Drosophila simulans* are more uniform across chromosomes than in *Drosophila melanogaster*. Red lines show the recombination rate in *D. simulans* for each of the major chromosomes (name labels in top margin), smoothed in this case at a 101-kb window size with a moving median. For comparison, blue lines show the recombination rate in *D. melanogaster* (with data taken from Comeron et al. [2012]); figure 3 for other resolutions.

**Fig. 3.**
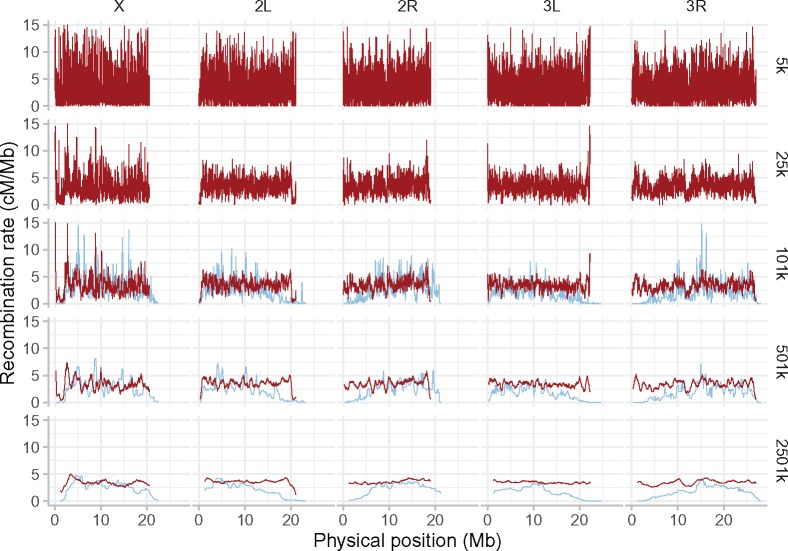
—Recombination rates in *Drosophila simulans* are more uniform across chromosomes than in *Drosophila melanogaster*, at all smoothing scales. Red lines show the recombination rate in *D. simulans* for each of the major chromosomes (names in top margin), smoothed now at five different window sizes (right margin, in bp) with a moving median. For comparison, blue lines show the recombination rate in *D. melanogaster* (data taken from Comeron et al. [2012] at 101 kb; and smoothed at 501 and 2,501 kb; with data not available at smaller resolutions).

We present the first high-resolution recombination map for *D. simulans*, and contrast it to that of *D. melanogaster* (Comeron et al. 2012). Across the range of smoothing parameters that we have applied to both species, the *D*. *simulans* recombination map is more uniform than that of *D*. *melanogaster* (figs. 2 and 3). The level of recombination suppression is lower toward the centromere in *D. simulans*. As in *D. melanogaster*, the main broad-scale features of the *D. simulans* map hold across the full range of genomic scales, whereas finer resolution peaks and troughs become visible only at higher resolutions, at the 5–501-kb scale (fig. 3). The finer-scale peaks (on a kb scale), as with the broader features (on a Mb scale), differ between these two sister *s*pecies, and persist across smoothing scales (see figs. 2 and 3).

### Motif Density Landscapes Are Similar in *D. simulans* and *D. melanogaster*

**Fig. 4.**
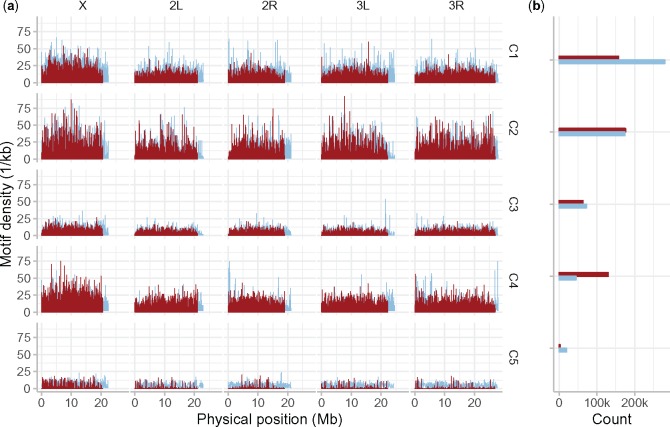
—Motif densities are similar in *Drosophila simulans* and *Drosophila melanogaster*. (*a*) Red (*D. simulans*) and blue (*D. melanogaster*) lines show motif densities across major chromosomes (top margin) as reported by FIMO, with motif occurrences discounted by 1 – *q* (see main text) and binned into 1-kb windows for each consensus motif (C1–5). (*b*) Total motif counts across all five large chromosomes. FIMO threshold: *P* value of 1e-4 (default setting).

We identify five consensus motifs based on motifs recovered in each of the two species (Motif Consensus Set section) and obtain their genome-wide densities. The consensus motifs were C1 = [A]_11_, C2 = [GCA]_4_, C3 = [CA]_6_, C4 = [TA]_5_, and C5 = [G]_8_, all five of which were significantly associated with recombination on at least one genomic resolution in *D. melanogaster* and present in both species, with C1–3 and C5 significantly associated at least once in *D. simulans*. Across all chromosomes and consensus motifs, the motif density landscapes were similar in *D. melanogaster* and *D. simulans* (fig. 4). This was especially true for intermediate size landscape features, such as humps and wider valleys (e.g., motif C2 on X, 7.5-Mb position, or 2L at the 8 and 12-Mb positions, fig. 4). Therefore, motif density cannot explain the differences in the broad recombination landscape between both species.

Finer resolution peaks and troughs varied more between species (e.g., motif C4 on X, 5–15-Mb position, fig. 4). Further, although the different motifs, C1–5, displayed similar broad patterns in each species—per chromosome and genome-wide—some species-specific patterns were seen. Motifs C1, [A]_11_ and C5, [G]_8_ were far less common in *D. simulans*, which had a lower total motif count, whereas the opposite was true for motif C4, [TA]_5_. Nonetheless, genome-wide motif distributions were similar in each species.

### Associations between Motif Densities and Recombination Rates Are Generally Weaker and Less Significant in *D. simulans* than in *D. melanogaster*

**Fig. 5.**
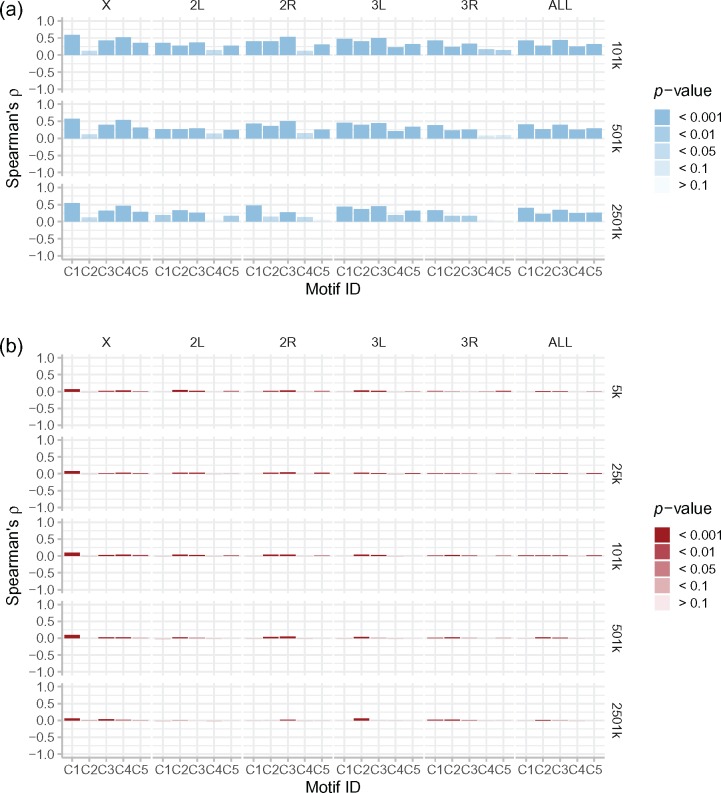
—Associations between motif densities and recombination rates are generally weaker and less significant in *Drosophila simulans* than in *Drosophila melanogaster*. For (*a*) *D. melanogaster*, and (*b*) *D. simulans*, bars indicate Spearman’s rho, *ρ*, (height) and the corresponding *P* value (transparency), from tests of the correlation between motif densities (as shown in fig. 4 for *D. simulans*, but rebinned for *D. melanogaster* to account for the resolution of the available data) and recombination rates, across individual chromosomes and for the five large chromosomes together (top margin), at all smoothing levels (see right margin).

We examined correlations between motif densities and recombination rates in each species, both per chromosome, and genome-wide, and at a range of genomic scales. A clear difference was observed between the species. In *D. melanogaster*, all but one correlation was positive, most were highly significant both genome-wide and per chromosome, and the correlation coefficients (Spearman’s *ρ*) were generally large; with a range of ∼0.4–0.6 for the most associated motifs per chromosome (and genome-wide, fig. 5*a*). In contrast, the associations observed in *D. simulans* were heterogeneously positive or negative, had lower significances than those observed in *D. melanogaster*, and were in all cases weak; with a range of ∼0.01–0.04 for the most associated motifs per chromosome (and genome-wide, fig. 5*b*). In both species, there was also variation in the importance of different motifs on different chromosomes (see below). However, although in *D. melanogaster*, the patterns of motif association held across all scales for each chromosome and genome-wide, in *D. simulans* there were occasional exceptions to this rule. For instance, on 2L, 2R, 3L, and genome-wide, the positive correlations for C1 and C4 switched direction at scales larger than 25–101 kb. Given that these correlations were very weak with low significance, we attribute these discrepancies stochastic noise, rather than biological signals. We finally note that motifs C1, C2, and C3 were the most associated with recombination across most major chromosomes in both species (though to a far lesser extent in *D. simulans*), but that an exception is observed for the X chromosome. Here, motif C2 had a very weak association with recombination rate in both species, and motif C4 instead had a high association, relative to its weak association on most autosomes in both species. Very similar observations were seen for the linear regressions (supplementary fig. S1, Supplementary Material online), with more models being significant and positive for *D. melanogaster*.

## Discussion

We present the first high-resolution recombination map for *D. simulans*, and a comparative analysis of recombination-associated motifs and their association with recombination in two sister species, *D. melanogaster* and *D. simulans*. We tested the hypothesis that such motifs predict recombination rates within the *D. melanogaster* species subgroup.

Our *D. simulans* recombination map confirms the results of previous, lower resolution work in this species (Stuktevanat 1929; Ohnishi and Voelker 1979, 1981; True et al. 1996). We find that the *D. simulans* recombination landscape is far flatter than in *D. melanogaster* (figs. 2 and 3). Although centromeric recombination suppression on the X, and to some extent on 2L and 3R, is observed in *D. simulans*, it is restricted to a small genomic region, whereas in *D. melanogaster* the recombination rate decreases only gradually over a much larger region in proximity to the centromeres (Comeron et al. 2012). In *D. simulans*, a similarly sharp teleomeric suppression is observed on 2L (and to some extent on X, 2R, 3L, and 3R) at most smoothing scales (this pattern is less clear at 2,501 kb). Unlike in *D. melanogaster* (Comeron et al. 2012), overall recombination rates in *D. simulans* appear quite similar between X and the autosomes (figs. 2 and 3).

We caution however, that recombination rate estimates from population polymorphism data are sensitive to demographic events, and particularly the ratio of X-chromosomal and autosomal variation differs widely between populations (Kauer et al. 2002; Schöfl and Schlötterer 2004). In *D. simulans* and *D. melanogaster*, mid-to-large-scale recombination features clearly persist over the 101, 501, and 2,501-kb smoothed maps. In *D. simulans*, our high-resolution map shows that such features also persist down to the 25 and 5-kb scale (e.g., the dip on 3R at 12.5-Mb position, fig. 3). As with the centromeric differences however, midscale and narrower landscape features differ between the species, especially at the 101 and 501-kb resolutions. In short, at all genomic scales tested the two species differ dramatically in recombination rates, over broad- and finer-scale recombination features.

One important difference between the recombination maps between the two species is that for *D. simulans*, we inferred the recombination landscape from population polymorphism data, whereas the recombination map of *D. melanogaster* is based on recombination events occurring in the laboratory. Because polymorphism patterns in natural populations are affected by selection and demography, this may impact the inferred recombination map. In particular the well-studied influence of recombination rate on the patterns of natural variation in positive (hitchhiking, Maynard Smith 1974; Hartfield and Otto 2011) and negative (background selection, Charlesworth et al. 1995; Comeron 2017) selection will also affect any polymorphism-based recombination maps. With regards to the inferred *D. simulans* recombination map, it is important to note that we inferred a rather similar recombination rate along the chromosomes, strongly suggesting very limited differences in *N*_e_, such that while our recombination map based on *ρ* will incorporate information about *N*_e_ (*ρ* = *N*_e_ × *r*) the minimal effects of variable linked selection along the chromosomes mean this is unlikely to produce biases between the maps used for *D. melanogaster* and *D. simulans* in this study. Hence, we consider population data based recombination map inference a reliable method, despite its obvious limitations.

Direct implications from these differences in genetic maps are that linkage disequilibrium should be both lower and less variable across the *D. simulans* chromosomes relative to those of *D. melanogaster*. It is important to keep in mind though that the *D. simulans* reference genome includes less repetitive DNA at the centromeric and telomeric ends of the chromosomes, so a comparison of recombination rates in not possible at the extremes of these regions. Nonetheless, our results bolster the current understanding of *D. simulans* recombination as less heterogeneous than that of *D. melanogaster* (True et al. 1996; Comeron et al. 2012), and indicate that selection will be generally more efficient in *D. simulans*, as genes that are uncoupled by recombination may result in more distinct signals of selection, in particular in Evolve and Resequence experiments (Kofler and Schlötterer 2014; Tobler et al. 2014; Barghi et al. 2017). Hence, adaptive evolutionary changes may occur more rapidly in *D. simulans*, all else being equal, because Hill-Robertson effects are reduced by the higher recombination (Hill and Robertson 1966).

Turning to the causes of this recombination variation, we ran a MEME motif search to identify short DNA sequence motifs associated with regions of higher than average recombination at a very coarse scale, repeating this search in both *D. melanogaster*, and *D. simulans*. The first point of note was that a larger number of motifs were returned in *D. melanogaster*, and that those in *D. simulans* were by tendency both shorter and showed a less significant association with recombination rate, with lower quality matches. Nonetheless, a generally similar set of motifs was recovered in each species, and across each recombination map smoothing scale investigated. In short, we obtained a subset of the *D. melanogaster* motifs in *D. simulans*; motifs C1, C2, C3, and C5, providing some confidence in the impact of these motifs on the recombination rate. The motif sharing between the two *Drosophila* species also provides some evidence that recombination motifs may to some degree be universal across *Drosophila* species. This idea builds upon prior work, which has shown that there is some overlap in motifs between more distant *Drosophila* species, such as *D. pseudoobscura*, which exhibits CACAC (Cirulli et al. 2007), CCCCACCCC, and CCTCCCT motifs (Kulathinal et al. 2008), and *D. persimilis*, which exhibits a CCNCCNTNNCCNC motif (Stevison and Noor 2010). This led Comeron et al. (2012) to speculate that *Drosophila* has a stable set of recombination motifs of universal function, which they confirmed in part by showing that *D. melanogaster* also exhibit the CACAC and CCTCCCT motifs, though not the CCCCACCCC motif. Our study builds on this result, showing that a larger degree of motif overlap can be seen both when contrasting consensus motifs and when comparing between more closely related species, and that the [CA]_*n*_ motif is universal to all *Drosophila* species studied. However, it is immediately notable that no complex, multipart motifs like CCNCCNTNNCCNC were recovered in our study.

The genome-wide distribution of motifs (fig. 4) revealed, somewhat surprisingly, that there are also clear parallels between the two species motif landscapes. Not only do motifs with higher density in *D. melanogaster* generally have a higher density in *D. simulans*, but the patterns of motif distribution genome-wide are also remarkably similar. For instance, a similar “hump” and “peak” can be observed at the 8 and 9-Mb positions of chromosomes X and 2L, respectively, for motif C2, in both species, whereas a density “trough” can be seen at 15 Mb on chromosome 2L for this motif (fig. 4). Motifs C1, C3, and C4 likewise exhibit very limited differences between species, on all chromosomes (fig. 4), despite clear differences in recombination rates (fig. 3). A few differences do exist. Motif C1 is more common in *D. melanogaster*, even if the “landscape” is similar to *D. simulans*; Motif C5 is then less common in *D. simulans*, and exhibits a distinct landscape on all autosomes, whereas any narrow-scale features rarely overlap between species, mirroring patterns of distinct recombination peaks and similar landscapes seen in *D. melanogaster* populations (Chan et al. 2012; Heil et al. 2015). Consequently, although it might be tempting to speculate that subtle differences in motif densities can explain the flatter recombination landscape of *D. simulans* and its unique recombination peak set, it is difficult to reconcile the distinctive patterns of recombination rate variation in the two species with their exceptionally similar motif density landscapes, that are almost identical between species, especially when focusing on the large-scale recombination differences that we observed in the centromeric and telomeric regions.

The similar motif density patterns between the two species cast doubt on the hypothesis that differences in motif distribution can account for differences in recombination variation in these species. If divergent motif densities really account for the species differences in recombination rates, how can we explain the discordance seen in the reduced recombination toward the centromeres in *D. melanogaster*, the lack of this reduction in *D. simulans*, and the similar motif distributions over these regions in both species? To investigate this observation quantitatively, we calculated Spearman’s rho, *ρ*, as an estimator of the correlation between genome-wide motif density and recombination rate (cM/Mb), for each motif, in each species, across a range of smoothing scales. This revealed a striking difference between the two species. In *D. melanogaster*, all associations (aside one) were positive, for all motifs at all scales tested, with low *P* values observed in most cases (fig. 5). These results accord well with those of Adrian et al. (2016), who found positive associations between motif densities and recombination rate in *D. melanogaster*, using a similar set of motifs (see supplementary S4, Supplementary Material online). In contrast, the associations observed in *D. simulans* were far smaller, and far more heterogeneous across chromosomes and motifs (fig. 5). This observation was confirmed by our linear regression models, fitted to explicitly test the predictive power of each motif to explain recombination rate variation, which showed an almost identical pattern to these correlations (supplementary fig. S1, Supplementary Material online). The correlational and model fit patterns were similar within each species across all smoothing scales, which is to say that the strength of the correlations observed did not increase or decrease with the higher or lower resolution of the recombination maps, at different scales. The clear implication is that motif densities do not universally predict recombination rates across the *Drosophila* clade, and are in particular not responsible for the large-scale differences observed between our two species. It is therefore pertinent to ask what alternative mechanisms could explain such differences.

A strong candidate is the dicistronic meiosis gene *mei-217*/*mei-218* and its protein product, MEI-218 (Brand et al. 2018), which is involved in the resolution of crossing over into DSBs and recombination (Brand et al. 2018). Divergent forms have recently been identified in *D. mauritiana* and *D. melanogaster*, species that diverged 0.6–0.9 Ma. Like *D. simulans*, *D. mauritiana* exhibits a higher and flatter recombination rate landscape than *D. melanogaster* (True et al. 1996), with the difference especially pronounced in the centromeric and telomeric regions (True et al. 1996), and with this pattern expressed to an even larger extent than is seen in *D. simulans* (True et al. 1996). Intriguingly then, Brand et al. (2018) also found a high divergence in DNA and protein structure in the *mei-217*/*mei-218* gene and MEI-218 protein between *D. mauritiana* and *D. melanogaster*. The *D. mauritiana* form was far more effective in promoting recombination, increasing recombination assurance and reducing crossover interference (Brand et al. 2018). It explained a large portion of the variance in crossover rates between *D. mauritiana* and *D. melanogaster*, especially that in the centromeric and telomeric regions (Brand et al. 2018), and so could be a primary mechanistic variant explaining the differences in recombination between *D. simulans* and *D. melanogaster*. The clear parallel differences between the recombination maps of *D. melanogaster* versus *D. mauritiana* and *D. melanogaster* versus *D. simulans* imply that *mei-217*/*mei-218* may be responsible for the heterogeneity in recombination landscape that we have observed, a possibility that should be explored and tested in future work.

What then might explain the clear correlations between motif density and recombination seen here in *D. melanogaster*, but not *D. simulans*? A simple explanation is that motifs are responsible for variation in recombination rate on a local scale. Hence, the lower density in *D. simulans* results also in less microscale variation in recombination rate. Alternatively, this pattern could be explained if the recombination motifs are recognized directly by cleavage proteins, similar to PRDM9 that differ in function or effectiveness between *D. simulans* and *D. melanogaster*. Recent evidence shows that a zinc-finger gene and protein of this type exists in *D. melanogaster* (Hunter, Huang, et al. 2016). Yet, such proteins tend to bind to complex, rather than short-repeat motifs, making this explanation unlikely. Another possibility relates to chromatin structure, because short-repeat DNA recombination motifs are thought to play roles in loosening chromatin structure, increasing access for DSB inducing proteins (Comeron et al. 2012; Adrian et al. 2016, and references therein). This could account for microvariation in recombination rates genome-wide between species, for instance because the motifs were generally shorter and so presumably less effective at chromatin loosening in *D. simulans*, genome-wide. Circumstantial evidence in favor of this hypothesis includes that in both species motif correlation patterns varied cross chromosomes – for instance, C4 was a good predictor only on X—suggesting that motifs can operate in a context dependent manner. Likewise, the removal of subcentromeric and subtelomeric region recombination data has been found not to alter correlational patterns in *D. melanogaster* (Adrian et al. 2016), suggesting that if motifs densities explain some recombination rate genome wide, they cannot explain centromeric and telomeric differences.

In short, we present the hypothesis that although short-repeat DNA motifs may affect recombination at a microscale, genome-wide, for instance in relation to euchromatic structure context, they cannot explain the large differences in recombination landscape differences between species, especially in the centromeric and telomeric regions. This variation seems far more likely to be explained by a mechanism such as *mei-217*/*mei-218*.

## Data Accessibility

Raw sequence reads for the 189 isofemale line haplotypes are available to download at the European Nucleotide Archive (ENA) under the primary and secondary accession numbers PRJEB29483 and ERP111789. Phased haplotypes are available from Dryad (doi:10.5061/dryad.744p394). Finally, CSV and MimicrEE ready text files for the *D. simulans* and *D. melanogaster* recombination maps are available at all resolutions from Dryad (doi:10.5061/dryad.744p394), as are the raw recombination rates output from LDJump in rho, *ρ*, before conversion to a cM/Mb scale (doi:10.5061/dryad.744p394), as well as motif density files, for genomic position and discounted motif count score (doi:10.5061/dryad.744p394).

## Supplementary Material

Supplementary data are available at *Genome Biology and Evolution* online.

## Supplementary Material

Supplementary Data
