# Supplementary material for: DNA Motifs Are Not General Predictors of Recombination in Two Drosophila Sister Species
Source: Genome Biol Evol. 2019 Apr 15;11(4):1345–57. doi: 10.1093/gbe/evz082 (PMC6490297; doi:10.1093/gbe/evz082)
Supplement: Supplementary Data [file evz082_supp.zip › X - Supporting Information Legends.docx]

**Supporting Information Legends**

**Fig. S1.** Linear models predict some of the variance in recombination rate in *D. melanogaster*, but not in *D. simulans*. Scatterplot of recombination rate vs. motif density for (a) *D. melanogaster* and (b) *D. simulans* (species also indicated by blue and red colour, respectively). Grey lines represent single-motif linear model fits, inset numbers the corresponding *r*^2^ values, and appended asterisks indicate the *p*-values of the model fits at * < 0.05, ** < 0.01, *** < 0.001. For purposes of this comparison only, smoothed *D. simulans* data at 101k is shown here, with the same resolution of the *D. melanogaster* data. The recognisable correlation features in (b) are unaffected by this downsampling step (not shown).

**Fig. S2.** LOESS-smoothed recombination maps. Red lines show the recombination rate in *D. simulans* for each of the major chromosomes (name labels in top margin), smoothed at 4 window sizes (see right margin, in bp) with the LOESS span parameter. LOESS span parameters correspond to 25, 101, 501, and 2501 kb, as span parameters equivalent to 5 kb can’t be implemented. For comparison, blue lines show the recombination rate in *D. melanogaster* (with data taken from Comeron, et al. 2012 at 101 kb; and then smoothed at 501 kb and 2501 kb; with the data for *D. melanogaster* not available at smaller resolutions).

**S3.** MEME motif discovery output for each *Drosophila* species at each genomic resolution.

**S4.** TomTom contrast of motifs from Adrian et al. (2016) to our set of 5 consensus motifs.

**S5.** R-Markdown document with script to reproduce our results (doi:10.5061/dryad.744p394).

**Table S1.** Pearson’s rho, *ρ*, for correlation between recombination rate and motif density, per motif, chromosome, genomic scale and species.
