# Supplementary material for: DNA Motifs Are Not General Predictors of Recombination in Two Drosophila Sister Species
Source: Genome Biol Evol. 2019 Apr 15;11(4):1345–57. doi: 10.1093/gbe/evz082 (PMC6490297; doi:10.1093/gbe/evz082)
Supplement: Supplementary Data [file evz082_supp.zip › SI.3.pdf]

Howie et al. Supplementary Information III [S3]

MEME motif discovery output for each *Drosophila* species at each genomic resolution.

MEME motif discovery output for *D. melanogaster*, 101kb windows.

MEME

Multiple Em for Motif Elicitation

For further information on how to interpret these results or to get a copy of the MEME software please access http://meme-suite.org.

If you use MEME in your research, please cite the following paper:
Timothy L. Bailey and Charles Elkan, "Fitting a mixture model by expectation maximization to discover motifs in biopolymers", *Proceedings of the Second International Conference on Intelligent Systems for Molecular Biology*, pp. 28-36, AAAI Press, Menlo Park, California, 1994. [pdf]

DISCOVERED MOTIFS | MOTIF LOCATIONS | INPUTS & SETTINGS | PROGRAM INFORMATION | RESULTS IN TEXT FORMAT NEW | RESULTS IN XML FORMAT NEW

DISCOVERED MOTIFS

		Logo	E-value
1.	+ -	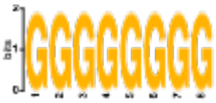	7.8e-034
2.	+ -	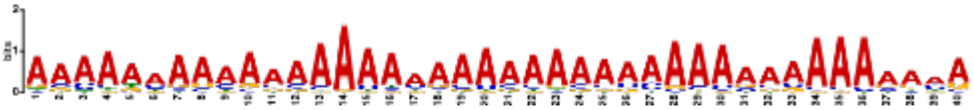	2.3e-043
3.	+ -	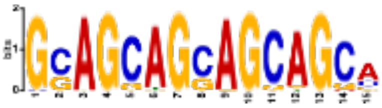	3.8e-030
4.	+ -	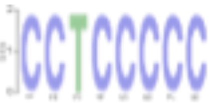	3.6e-001
5.	+ -	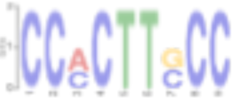	6.5e+000
6.	+ -	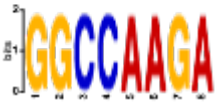	7.1e-009
7.	+ -	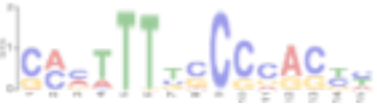	6.1e-001
8.	+ -	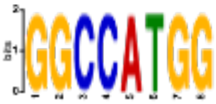	1.0e-004
9.	+ -	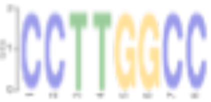	1.7e+000
10.	+ -	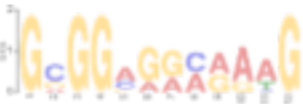	4.8e+000

Stopped because requested number of motifs (10) found.

MOTIF LOCATIONS

(•) Only Motif Sites ( ) Motif Sites+Scanned Sites ( ) All Sequences

Name Location	*p*-value	Motif Location
1. X:2300001-2400000	1.53e-7	+ -
2. X:2400001-2500000	1.75e-6	+ -
3. X:2600001-2700000	2.22e-7	+ -
4. X:2900001-3000000	9.40e-8	+ -
5. X:3000001-3100000	4.88e-11	+ -
6. X:3200001-3300000	3.57e-10	+ -
7. X:3300001-3400000	6.62e-9	+ -
8. X:3500001-3600000	2.19e-6	+ -
9. X:3600001-3700000	2.08e-9	+ -
10. X:3800001-3900000	1.29e-10	+ -
11. X:3900001-4000000	4.85e-8	+ -
12. X:4100001-4200000	3.21e-7	+ -
13. X:4300001-4400000	1.65e-11	+ -
14. X:4400001-4500000	1.52e-8	+ -
15. X:4500001-4600000	5.01e-9	+ -
16. X:4600001-4700000	1.12e-8	+ -
17. X:4700001-4800000	2.53e-6	+ -
18. X:4800001-4900000	4.06e-7	+ -
19. X:4900001-5000000	3.25e-10	+ -
20. X:5000001-5100000	6.03e-11	+ -
21. X:5100001-5200000	4.62e-11	+ -
22. X:5200001-5300000	3.42e-9	+ -
23. X:5300001-5400000	7.55e-9	+ -
24. X:5500001-5600000	2.10e-8	+ -
25. X:5600001-5700000	3.11e-7	+ -
26. X:5700001-5800000	7.04e-7	+ -
27. X:6100001-6200000	1.98e-7	+ -
28. X:6300001-6400000	3.86e-9	+ -
29. X:6400001-6500000	1.45e-6	+ -
30. X:6500001-6600000	9.20e-11	+ -
31. X:6600001-6700000	3.20e-11	+ -
32. X:6700001-6800000	2.54e-8	+ -
33. X:6800001-6900000	6.57e-6	+ -

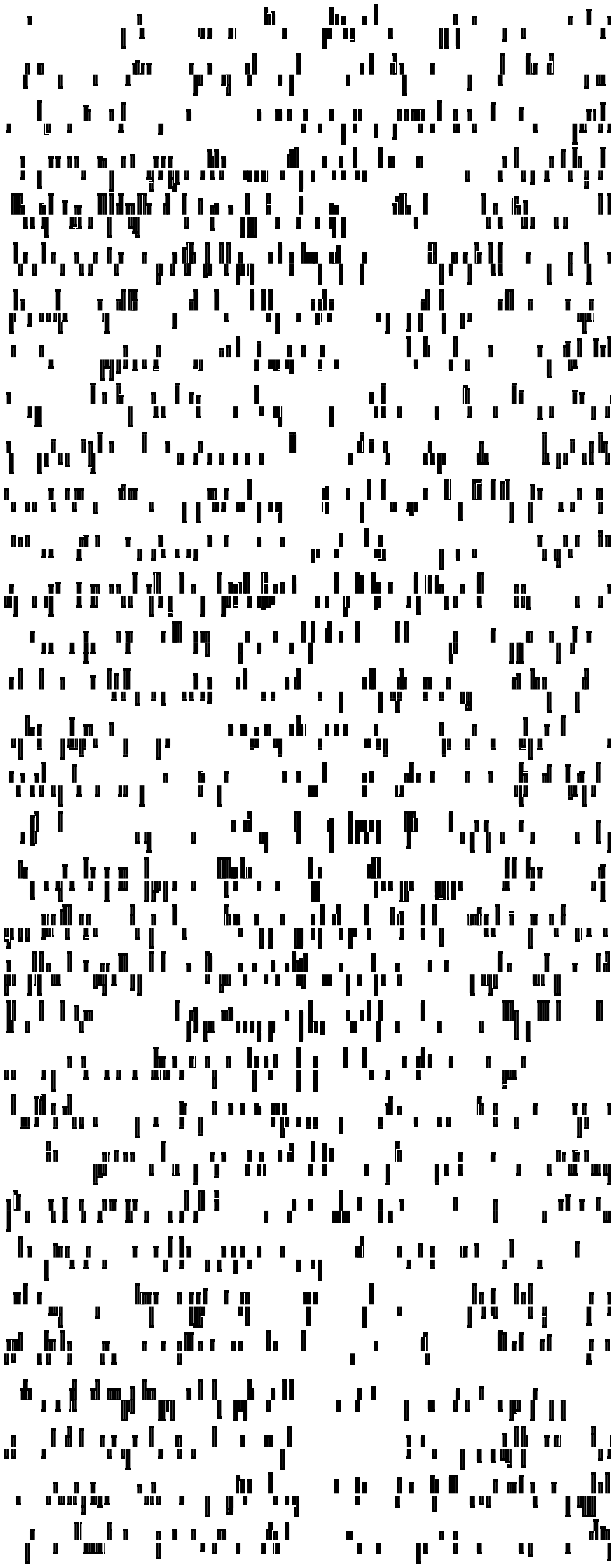

34.	X:6900001-7000000	1.42e-9
35.	X:7600001-7700000	3.62e-7
36.	X:8100001-8200000	2.04e-7
37.	X:8200001-8300000	3.52e-8
38.	X:8300001-8400000	9.16e-14
39.	X:8400001-8500000	5.14e-9
40.	X:8500001-8600000	7.70e-8
41.	X:8600001-8700000	6.65e-9
42.	X:8700001-8800000	4.15e-9
43.	X:8800001-8900000	7.90e-6
44.	X:8900001-9000000	1.02e-7
45.	X:9000001-9100000	1.07e-8
46.	X:9100001-9200000	2.58e-7
47.	X:9200001-9300000	6.02e-8
48.	X:9300001-9400000	1.59e-8
49.	X:9400001-9500000	3.27e-6
50.	X:9500001-9600000	6.93e-6
51.	X:9600001-9700000	3.36e-8
52.	X:9700001-9800000	1.17e-6
53.	X:9900001-10000000	6.28e-7
54.	X:10100001-10200000	6.60e-9
55.	X:10200001-10300000	2.33e-14
56.	X:10800001-10900000	1.15e-6
57.	X:10900001-11000000	1.92e-8
58.	X:11000001-11100000	2.27e-8
59.	X:11100001-11200000	5.49e-10
60.	X:11200001-11300000	9.21e-9
61.	X:11800001-11900000	5.83e-8
62.	X:11900001-12000000	6.75e-7
63.	X:12000001-12100000	3.20e-8
64.	X:12200001-12300000	5.09e-7
65.	X:12500001-12600000	2.00e-7
66.	X:12600001-12700000	6.70e-8
67.	X:12700001-12800000	1.76e-8
68.	X:12800001-12900000	1.22e-7
69.	X:12900001-13000000	1.08e-6

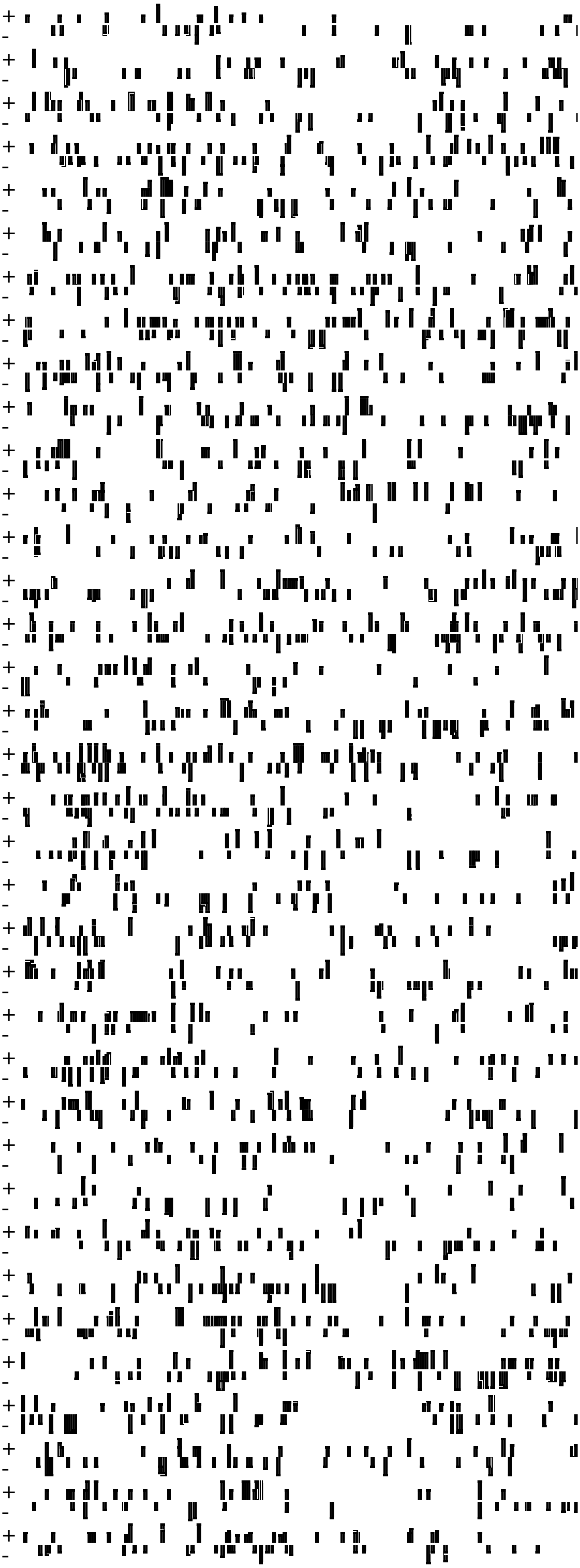

70.	X:13000001-13100000	8.39e-8
71.	X:13100001-13200000	4.42e-7
72.	X:13200001-13300000	2.02e-9
73.	X:13300001-13400000	1.13e-9
74.	X:13400001-13500000	1.21e-6
75.	X:13600001-13700000	2.18e-7
76.	X:13700001-13800000	6.38e-8
77.	X:13800001-13900000	8.15e-9
78.	X:13900001-14000000	9.17e-7
79.	X:14000001-14100000	6.79e-6
80.	X:14100001-14200000	6.65e-7
81.	X:14300001-14400000	5.05e-8
82.	X:14500001-14600000	7.35e-9
83.	X:14600001-14700000	3.98e-13
84.	X:14700001-14800000	1.96e-7
85.	X:15000001-15100000	8.87e-8
86.	X:15100001-15200000	1.45e-7
87.	X:15500001-15600000	7.01e-7
88.	X:15600001-15700000	6.40e-4
89.	X:15700001-15800000	1.83e-7
90.	X:16000001-16100000	5.74e-15
91.	X:16100001-16200000	6.59e-8
92.	X:16200001-16300000	6.75e-3
93.	X:16400001-16500000	4.90e-7
94.	X:16500001-16600000	1.06e-5
95.	X:16600001-16700000	1.11e-8
96.	X:16700001-16800000	6.15e-6
97.	X:16800001-16900000	1.69e-5
98.	X:17200001-17300000	8.64e-6
99.	X:17600001-17700000	1.35e-7
100.	X:17800001-17900000	7.91e-7
101.	X:18600001-18700000	2.51e-6
102.	X:19100001-19200000	1.44e-5
103.	X:19200001-19300000	8.68e-11
104.	X:19400001-19500000	1.04e-4
105.	X:19600001-19700000	2.39e-10

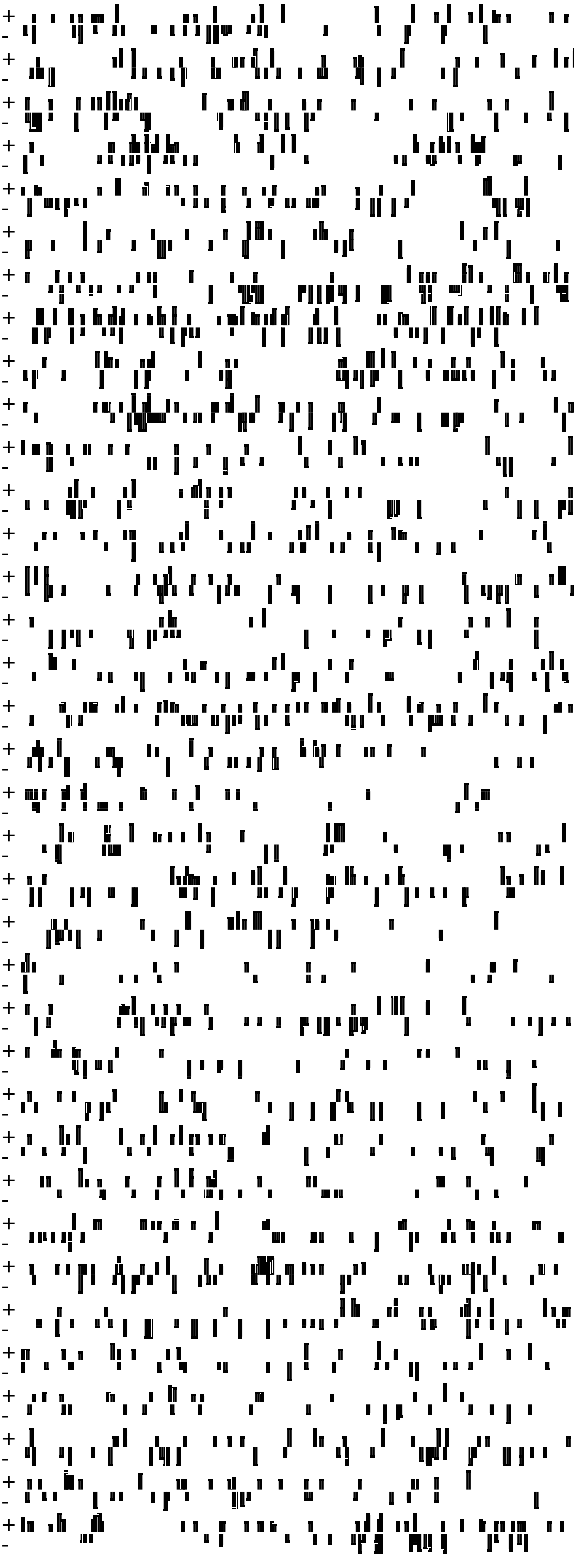

106.	X:19700001-19800000	1.29e-8	+ - 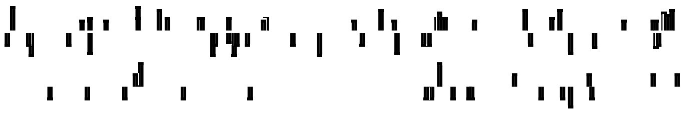
107.	X:19800001-19900000	5.48e-5	+ -

Inputs & Settings

Sequences

Role	Source	Alphabet	Sequence Count	Total Size
Primary Sequences	06DiscoMo/Dmel-101k-hi.fa	DNA	580	57999420
Control Sequences	06DiscoMo/Dmel-101k-lo.fa	DNA	614	61029075

Background Model

Source: the file '01MarkovModel/dmel5.36-clean.model'

Order: 0

Name	Freq.	Bg.				Bg.	Freq.	Name
Adenine	0.287	0.288	A	~	T	0.288	0.287	Thymine
Cytosine	0.213	0.212	C	~	G	0.212	0.213	Guanine

Other Settings

**Motif Site Distribution**	ANR: Any number of sites per sequence
**Objective Function**	Differential Enrichment mHG
**Starting Point Function**	log likelihood ratio (LLR)
**Site Strand Handling**	Sites may be on either strand
**Maximum Number of Motifs**	10
**Motif E-value Threshold**	no limit
**Minimum Motif Width**	8
**Maximum Motif Width**	50
**Minimum Sites per Motif**	1000
**Maximum Sites per Motif**	2900
**Bias on Number of Sites**	0.8
**Sequence Prior**	Simple Dirichlet
**Sequence Prior Source**	the file '01MarkovModel/dmel5.36-clean.model'
**Sequence Prior Strength**	0.01
**EM Starting Point Source**	From substrings in input sequences
**EM Starting Point Map Type**	Uniform
**EM Starting Point Fuzz**	0.5
**EM Maximum Iterations**	50
**EM Improvement Threshold**	0.00001
**Maximum Search Size**	100000

Hide Advanced Settings

MEME version
5.0.1 (Release date: Thu Jul 26 17:15:19 2018 -0700)

Reference

Timothy L. Bailey and Charles Elkan, "Fitting a mixture model by expectation maximization to discover motifs in biopolymers", *Proceedings of the Second International Conference on Intelligent Systems for Molecular Biology*, pp. 28-36, AAAI Press, Menlo Park, California, 1994.

Command line

```
meme -oc 07MemeDmel-101k -objfun de -neg 06DiscoMo/Dmel-101k-lo.fa -dna -revcomp
-mod anr -nmotifs 10 -minsites 1000 -bfile 01MarkovModel/dmel5.36-clean.model
06DiscoMo/Dmel-101k-hi.fa
```

MEME motif discovery output for *D. melanogaster*, 501kb windows.

MEME

Multiple Em for Motif Elicitation

For further information on how to interpret these results or to get a copy of the MEME software please access http://meme-suite.org.

If you use MEME in your research, please cite the following paper:
Timothy L. Bailey and Charles Elkan, "Fitting a mixture model by expectation maximization to discover motifs in biopolymers", *Proceedings of the Second International Conference on Intelligent Systems for Molecular Biology*, pp. 28-36, AAAI Press, Menlo Park, California, 1994. [pdf]

DISCOVERED MOTIFS | MOTIF LOCATIONS | INPUTS & SETTINGS | PROGRAM INFORMATION | RESULTS IN TEXT FORMAT NEW | RESULTS IN XML FORMAT NEW

DISCOVERED MOTIFS

Logo

1. + -
2. + -
3. + -
4. + -
5. + -
6. + -
7. + -
8. + -
9. + -
10. + -

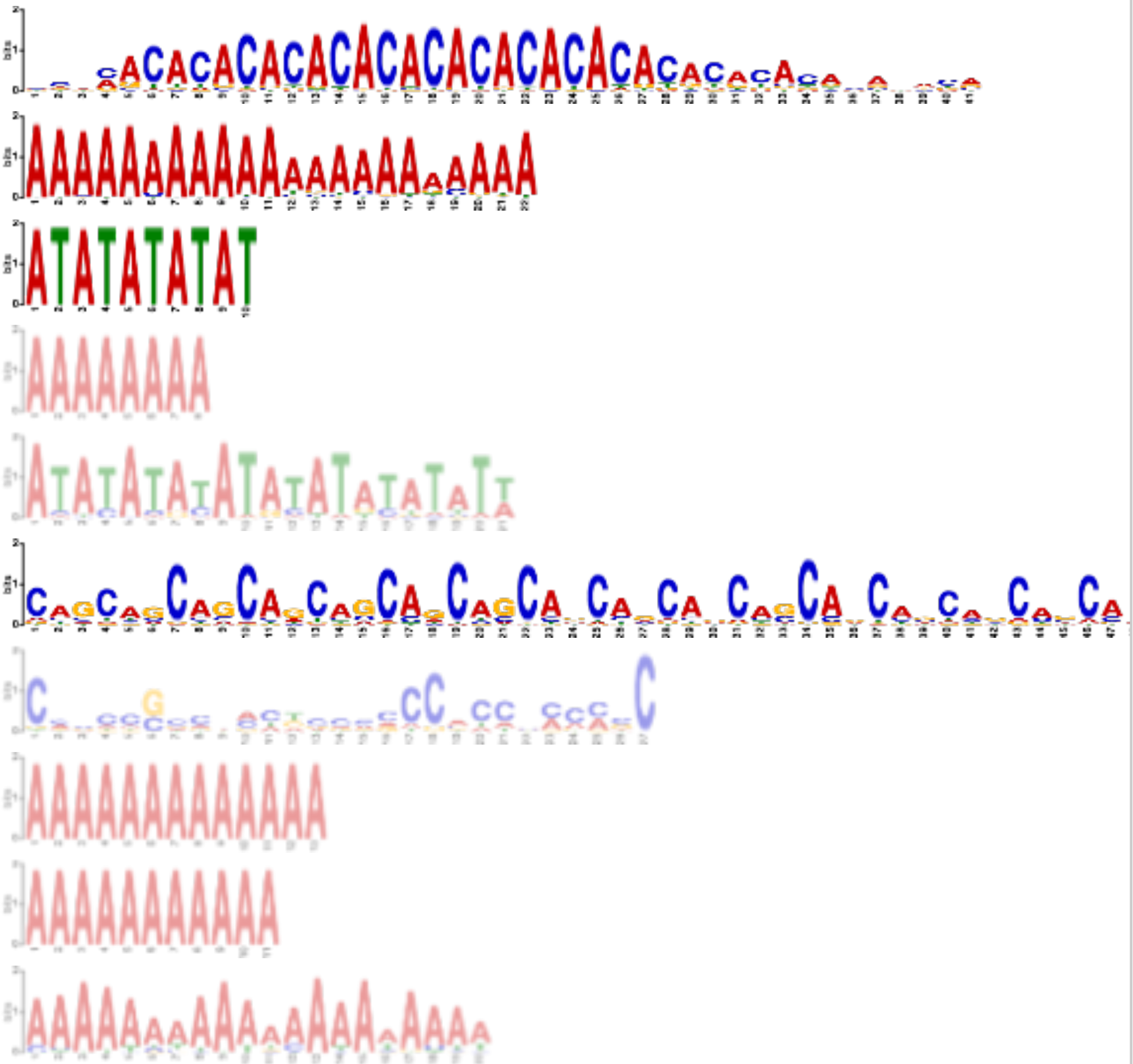

Stopped because requested number of motifs (10) found.

Motif Locations

Only Motif Sites   Motif Sites+Scanned Sites   All Sequences

	Name	*p*-value	Motif Location
1.	X:3000001-3100000	1.23e-49	
2.	X:3100001-3200000	1.46e-43	
3.	X:3200001-3300000	1.42e-48	
4.	X:3300001-3400000	6.73e-44	
5.	X:3400001-3500000	2.56e-42	
6.	X:3700001-3800000	4.21e-46	
7.	X:3900001-4000000	1.63e-50	
8.	X:4000001-4100000	1.54e-31	
9.	X:4100001-4200000	5.36e-32	
10.	X:4200001-4300000	4.64e-32	
11.	X:4300001-4400000	1.64e-39	
12.	X:4400001-4500000	1.19e-40	
13.	X:4500001-4600000	1.57e-45	
14.	X:4600001-4700000	1.54e-46	
15.	X:4700001-4800000	5.51e-45	
16.	X:4800001-4900000	4.83e-38	
17.	X:4900001-5000000	2.44e-38	
18.	X:5000001-5100000	7.18e-37	
19.	X:5100001-5200000	1.64e-43	
20.	X:5200001-5300000	8.08e-39	
21.	X:5300001-5400000	2.51e-39	
22.	X:5400001-5500000	1.10e-47	
23.	X:5500001-5600000	4.04e-37	
24.	X:5600001-5700000	2.85e-43	
25.	X:5700001-5800000	9.38e-38	
26.	X:6200001-6300000	5.28e-38	
27.	X:6300001-6400000	8.64e-36	
28.	X:6400001-6500000	2.51e-40	
29.	X:6500001-6600000	2.36e-42	
30.	X:6600001-6700000	3.41e-35	
31.	X:6700001-6800000	9.47e-45	
32.	X:6800001-6900000	3.45e-43	
33.	X:6900001-7000000	5.13e-34	

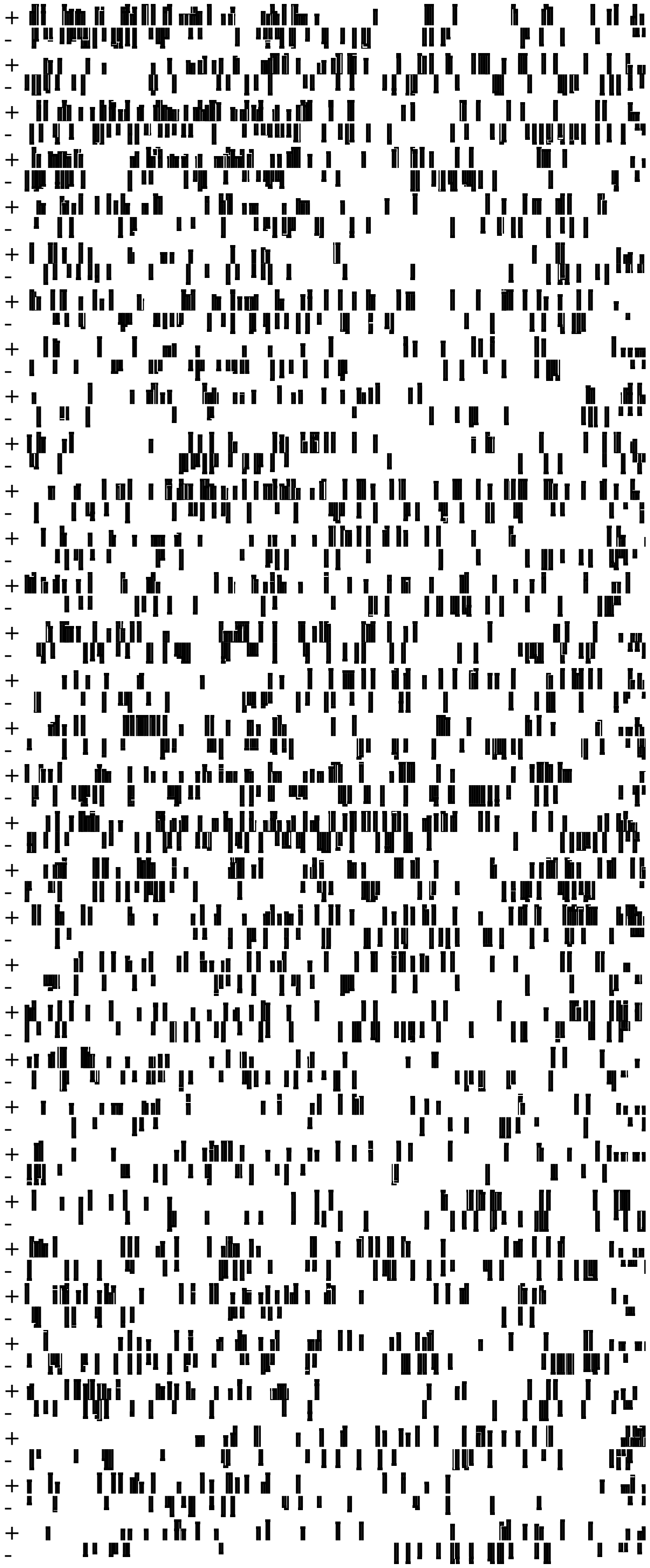

34.	X:8100001-8200000	8.73e-40
35.	X:8200001-8300000	4.25e-42
36.	X:8300001-8400000	5.47e-40
37.	X:8400001-8500000	2.19e-34
38.	X:8500001-8600000	5.67e-41
39.	X:8600001-8700000	4.22e-43
40.	X:8700001-8800000	1.36e-44
41.	X:8800001-8900000	2.90e-39
42.	X:8900001-9000000	2.99e-42
43.	X:9000001-9100000	1.67e-37
44.	X:9100001-9200000	1.28e-29
45.	X:9200001-9300000	7.04e-42
46.	X:9300001-9400000	1.73e-40
47.	X:9400001-9500000	3.26e-34
48.	X:9500001-9600000	1.04e-40
49.	X:9600001-9700000	5.63e-45
50.	X:9700001-9800000	1.01e-45
51.	X:9800001-9900000	1.02e-34
52.	X:9900001-10000000	3.42e-33
53.	X:10000001-10100000	1.46e-38
54.	X:10100001-10200000	5.25e-44
55.	X:10800001-10900000	4.46e-42
56.	X:10900001-11000000	1.18e-28
57.	X:11000001-11100000	1.04e-40
58.	X:11100001-11200000	1.56e-39
59.	X:11200001-11300000	7.57e-39
60.	X:11800001-11900000	1.67e-26
61.	X:11900001-12000000	1.61e-44
62.	X:12000001-12100000	8.29e-42
63.	X:12500001-12600000	7.94e-43
64.	X:12600001-12700000	6.71e-52
65.	X:12700001-12800000	6.32e-47
66.	X:12800001-12900000	1.72e-40
67.	X:12900001-13000000	1.10e-33
68.	X:13000001-13100000	6.15e-39
69.	X:13100001-13200000	2.48e-43

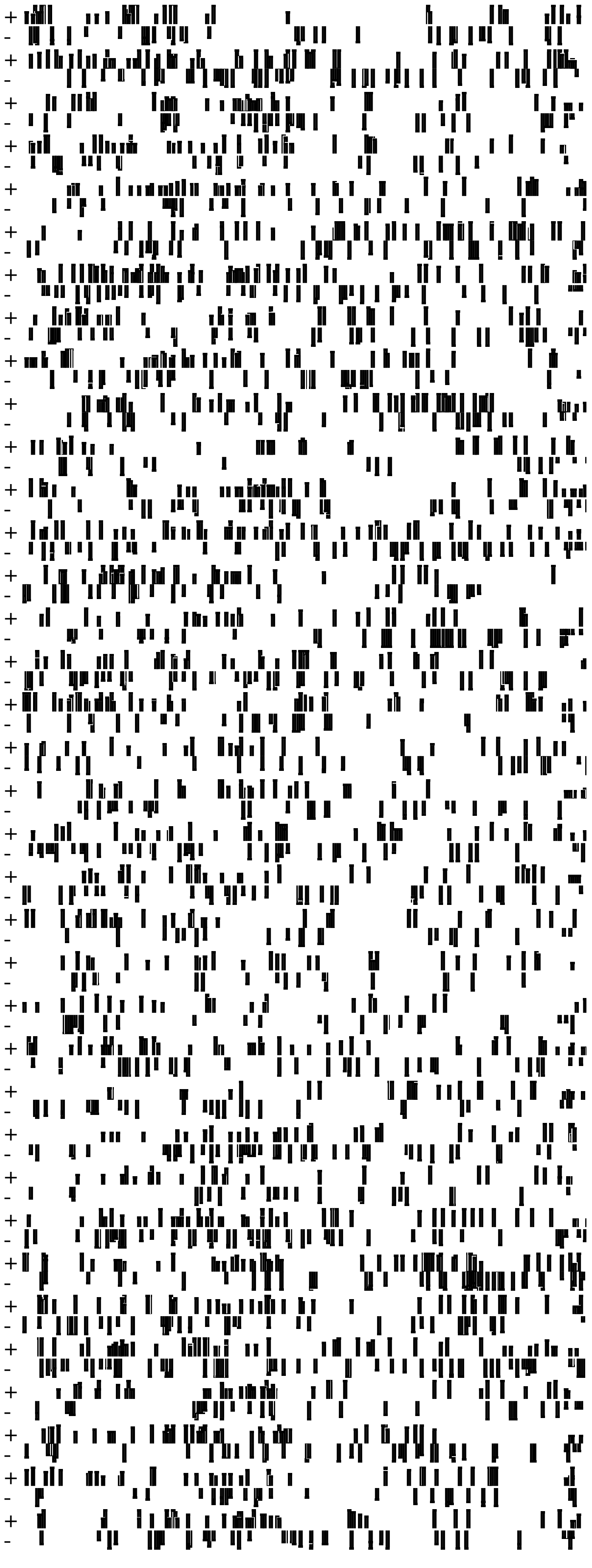

70.	X:13200001-13300000	5.96e-36
71.	X:13300001-13400000	4.94e-42
72.	X:13400001-13500000	5.64e-41
73.	X:13500001-13600000	1.82e-38
74.	X:13600001-13700000	2.52e-29
75.	X:13700001-13800000	2.57e-47
76.	X:13800001-13900000	4.24e-50
77.	X:13900001-14000000	9.13e-42
78.	X:14000001-14100000	7.91e-42
79.	X:14100001-14200000	8.98e-36
80.	X:14200001-14300000	1.48e-32

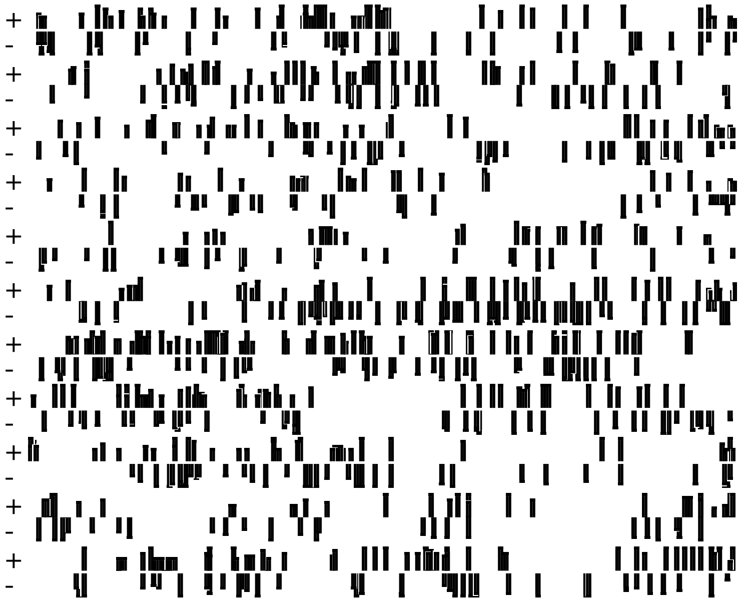

INPUTS & SETTINGS

Sequences

Role	Source	Alphabet	Sequence Count	Total Size
Primary Sequences	06DiscoMo/Dmel-501k-hi.fa	DNA	547	54699453
Control Sequences	06DiscoMo/Dmel-501k-lo.fa	DNA	647	64329042

Background Model

Source: the file '01MarkovModel/dmel5.36-clean.model'

Order: 0

Name	Freq.	Bg.				Bg.	Freq.	Name
Adenine	0.287	0.288	A	~	T	0.288	0.287	Thymine
Cytosine	0.213	0.212	C	~	G	0.212	0.213	Guanine

Other Settings

**Motif Site Distribution**	ANR: Any number of sites per sequence
**Objective Function**	Differential Enrichment mHG
**Starting Point Function**	log likelihood ratio (LLR)
**Site Strand Handling**	Sites may be on either strand
**Maximum Number of Motifs**	10
**Motif E-value Threshold**	no limit
**Minimum Motif Width**	8
**Maximum Motif Width**	50
**Minimum Sites per Motif**	1000
**Maximum Sites per Motif**	2735

Show Advanced Settings

MEME version

5.0.1 (Release date: Thu Jul 26 17:15:19 2018 -0700)

Reference

Timothy L. Bailey and Charles Elkan, "Fitting a mixture model by expectation maximization to discover motifs in biopolymers", *Proceedings of the Second International Conference on Intelligent Systems for Molecular Biology*, pp. 28-36, AAAI Press, Menlo Park, California, 1994.

Command line

```
meme -oc 07MemeDmel-501k -objfun de -neg 06DiscoMo/Dmel-501k-lo.fa -dna -revcomp
-mod anr -nmotifs 10 -minsites 1000 -bfile 01MarkovModel/dmel5.36-clean.model
06DiscoMo/Dmel-501k-hi.fa
```

MEME motif discovery output for *D. melanogaster*, 2501kb windows.

MEME

Multiple Em for Motif Elicitation

For further information on how to interpret these results or to get a copy of the MEME software please access http://meme-suite.org.

If you use MEME in your research, please cite the following paper:
Timothy L. Bailey and Charles Elkan, "Fitting a mixture model by expectation maximization to discover motifs in biopolymers", *Proceedings of the Second International Conference on Intelligent Systems for Molecular Biology*, pp. 28-36, AAAI Press, Menlo Park, California, 1994. [pdf]

DISCOVERED MOTIFS | MOTIF LOCATIONS | INPUTS & SETTINGS | PROGRAM INFORMATION | RESULTS IN TEXT FORMAT NEW | RESULTS IN XML FORMAT NEW

DISCOVERED MOTIFS

		Logo	E-
1.	+ -	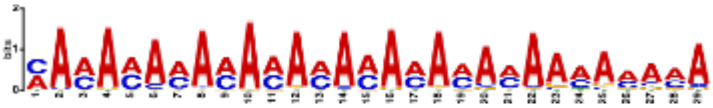	1
2.	+ -	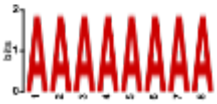	7
3.	+ -	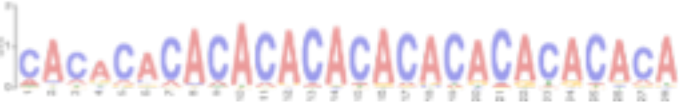	1
4.	+ -	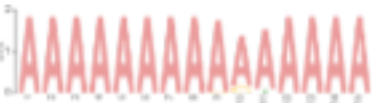	3.
5.	+ -	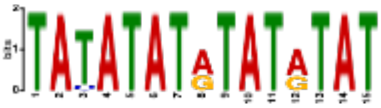	3
6.	+ -	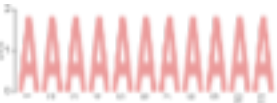	7.
7.	+ -	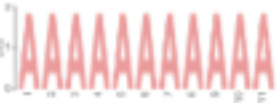	7.
8.	+ -	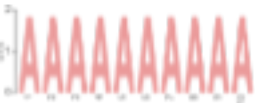	7.
9.	+ -	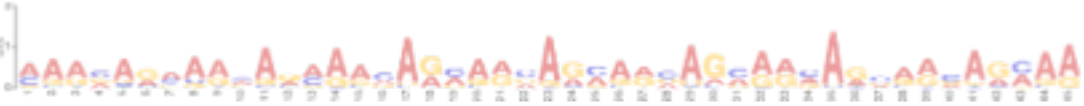	5.
10.	+ -	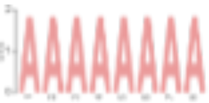	7.

Stopped because requested number of motifs (10) found.

Motif Locations

Only Motif Sites | Motif Sites+Scanned Sites | All Sequences

	Name	*p*-value
1.	X:3400001-3500000	4.21e-31
2.	X:3500001-3600000	8.04e-27
3.	X:3600001-3700000	3.88e-28
4.	X:3700001-3800000	2.74e-26
5.	X:3800001-3900000	2.60e-23
6.	X:3900001-4000000	1.35e-29
7.	X:4000001-4100000	3.06e-20
8.	X:4100001-4200000	6.98e-21
9.	X:4200001-4300000	9.76e-22
10.	X:4300001-4400000	2.44e-25
11.	X:4400001-4500000	1.97e-22
12.	X:4500001-4600000	3.70e-23
13.	X:4600001-4700000	8.71e-31
14.	X:4700001-4800000	1.51e-26
15.	X:4800001-4900000	2.74e-25
16.	X:4900001-5000000	3.02e-25
17.	X:5000001-5100000	3.92e-26
18.	X:5100001-5200000	4.19e-27
19.	X:5200001-5300000	3.34e-23
20.	X:5300001-5400000	6.71e-24
21.	X:5400001-5500000	3.31e-28
22.	X:5500001-5600000	4.82e-23
23.	X:5600001-5700000	8.89e-25
24.	X:5700001-5800000	3.51e-27
25.	X:5800001-5900000	1.25e-28
26.	X:5900001-6000000	2.43e-17
27.	X:6000001-6100000	3.46e-24
28.	X:6100001-6200000	4.47e-21
29.	X:6200001-6300000	1.71e-22
30.	X:6300001-6400000	5.20e-25
31.	X:6400001-6500000	6.47e-20
32.	X:6500001-6600000	1.30e-27
33.	X:7200001-7300000	1.09e-29

Motif Location

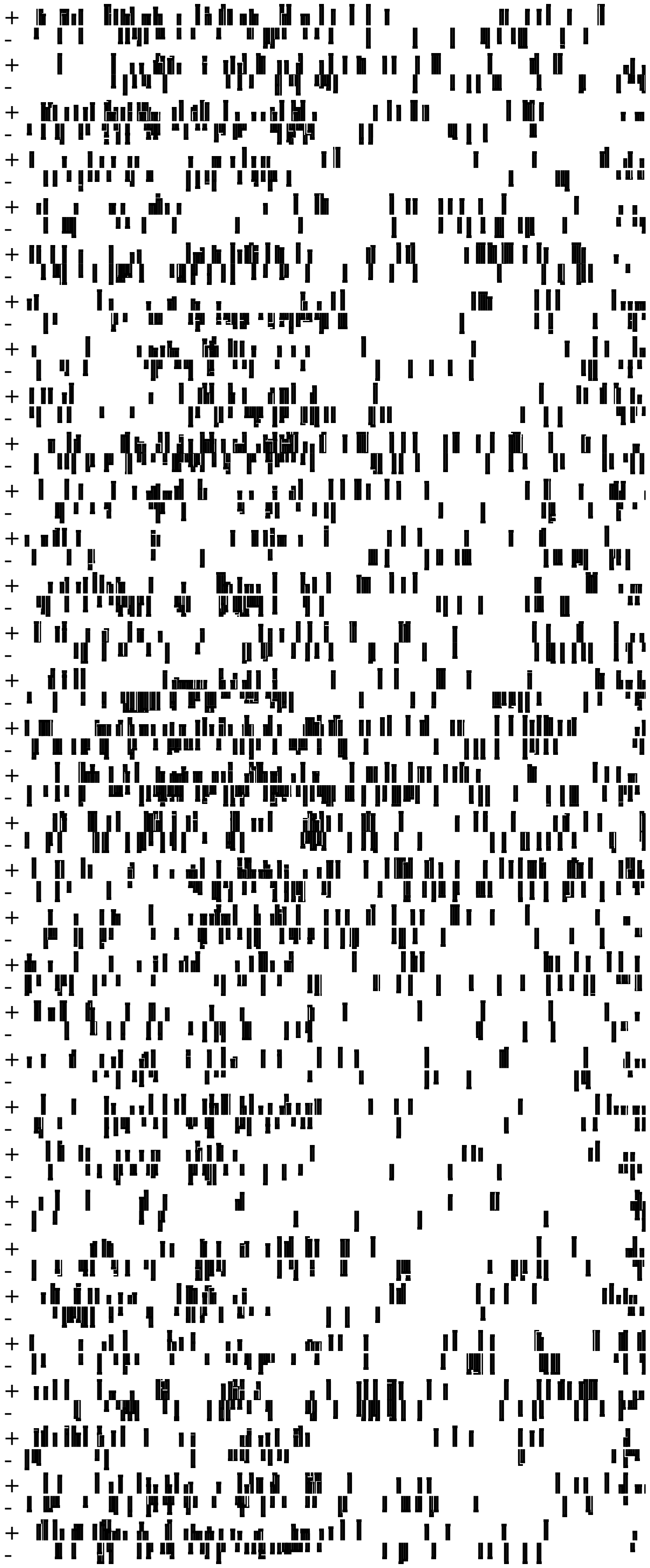

34.	X:7300001-7400000	9.53e-19
35.	X:7400001-7500000	3.01e-23
36.	X:7500001-7600000	1.84e-26
37.	X:7600001-7700000	2.41e-26
38.	X:7700001-7800000	1.29e-20
39.	X:7800001-7900000	2.83e-24
40.	X:7900001-8000000	5.59e-27
41.	X:8000001-8100000	1.26e-22
42.	X:8100001-8200000	4.67e-25
43.	X:8200001-8300000	8.27e-27
44.	X:8300001-8400000	9.98e-25
45.	X:8400001-8500000	1.10e-24
46.	X:8500001-8600000	2.08e-21
47.	X:8600001-8700000	8.37e-26
48.	X:8700001-8800000	1.73e-27
49.	X:8800001-8900000	9.11e-27
50.	X:8900001-9000000	5.79e-31
51.	X:9000001-9100000	6.44e-24
52.	X:9100001-9200000	3.01e-21
53.	X:9200001-9300000	1.89e-20
54.	X:9300001-9400000	5.55e-25
55.	X:9400001-9500000	9.05e-20
56.	X:9500001-9600000	7.25e-26
57.	X:9600001-9700000	1.05e-25
58.	X:9700001-9800000	1.02e-22
59.	X:9800001-9900000	1.88e-25
60.	X:9900001-10000000	2.02e-20
61.	X:10000001-10100000	1.27e-22
62.	X:10100001-10200000	1.48e-29
63.	X:10200001-10300000	3.68e-25
64.	X:10300001-10400000	3.52e-24
65.	X:10400001-10500000	1.08e-17
66.	X:10500001-10600000	9.10e-29
67.	X:10600001-10700000	8.15e-26
68.	X:10700001-10800000	3.79e-21
69.	X:10800001-10900000	1.69e-26

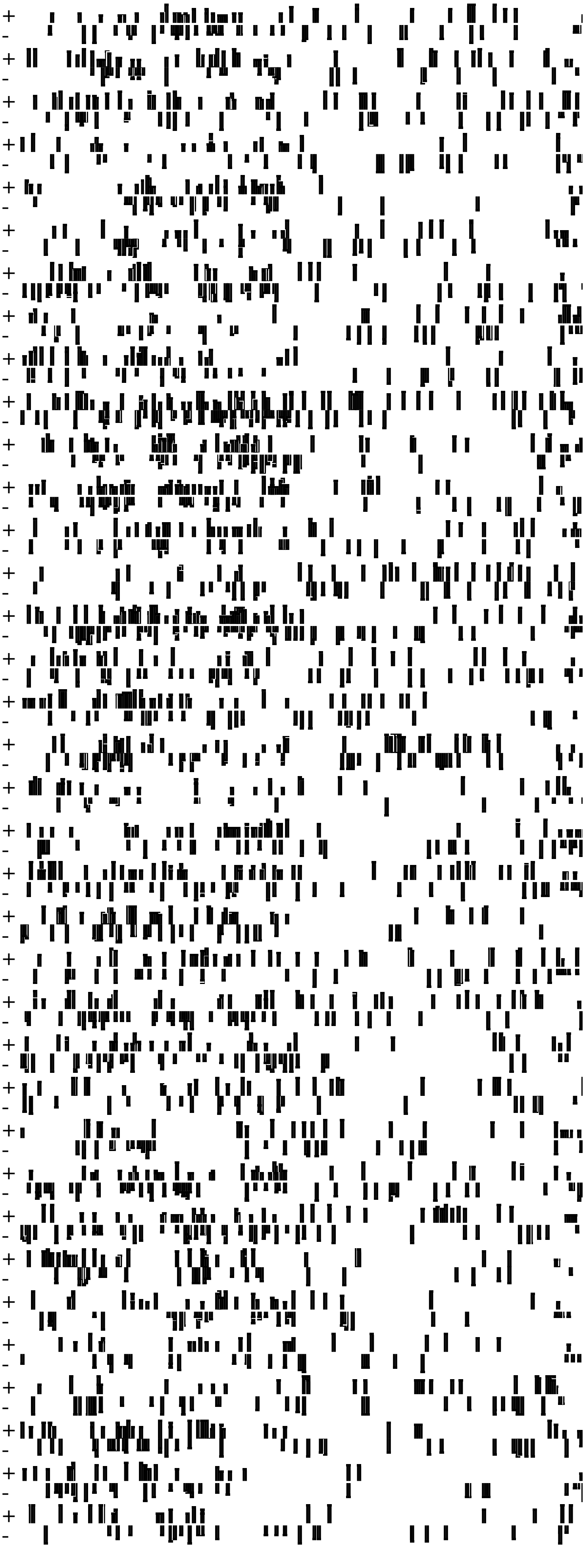

70.	X:11700001-11800000	1.01e-21
71.	X:11800001-11900000	1.30e-15
72.	X:11900001-12000000	1.99e-29
73.	X:12000001-12100000	1.06e-29
74.	X:12100001-12200000	9.29e-22
75.	X:12200001-12300000	1.92e-20
76.	X:12300001-12400000	7.89e-26
77.	X:12400001-12500000	1.63e-22
78.	X:12500001-12600000	1.71e-27
79.	X:12600001-12700000	5.29e-29
80.	X:12700001-12800000	2.36e-24

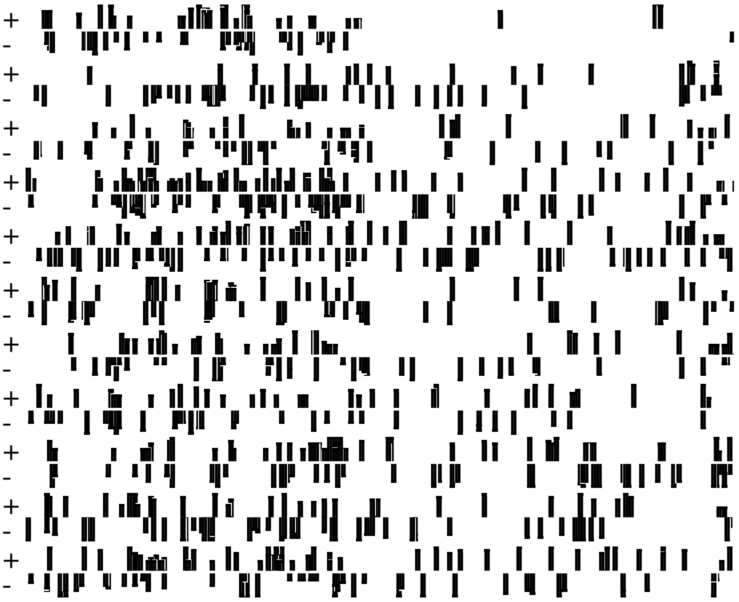

Inputs & Settings

Sequences

Role	Source	Alphabet	Sequence Count	Total Size
Primary Sequences	06DiscoMo/Dmel-2501k-hi.fa	DNA	568	56799432
Control Sequences	06DiscoMo/Dmel-2501k-lo.fa	DNA	626	62229063

Background Model

Source: the file '01MarkovModel/dmel5.36-clean.model'

Order: 0

Name	Freq.	Bg.				Bg.	Freq.	Name
Adenine	0.287	0.288	A	~	T	0.288	0.287	Thymine
Cytosine	0.213	0.212	C	~	G	0.212	0.213	Guanine

Other Settings

**Motif Site Distribution**	ANR: Any number of sites per sequence
**Objective Function**	Differential Enrichment mHG
**Starting Point Function**	log likelihood ratio (LLR)
**Site Strand Handling**	Sites may be on either strand
**Maximum Number of Motifs**	10
**Motif E-value Threshold**	no limit
**Minimum Motif Width**	8
**Maximum Motif Width**	50
**Minimum Sites per Motif**	1000
**Maximum Sites per Motif**	2840

Show Advanced Settings

MEME version

5.0.1 (Release date: Thu Jul 26 17:15:19 2018 -0700)

Reference

Timothy L. Bailey and Charles Elkan, "Fitting a mixture model by expectation maximization to discover motifs in biopolymers", *Proceedings of the Second International Conference on Intelligent Systems for Molecular Biology*, pp. 28-36, AAAI Press, Menlo Park, California, 1994.

Command line

```
meme -oc 07MemeDmel-2501k -objfun de -neg 06DiscoMo/Dmel-2501k-lo.fa -dna
-revcomp -mod anr -nmotifs 10 -minsites 1000 -bfile 01MarkovModel/dmel5.36-
clean.model 06DiscoMo/Dmel-2501k-hi.fa
```

MEME motif discovery output for *D. simulans*, 1kb windows.

MEME

Multiple Em for Motif Elicitation

For further information on how to interpret these results or to get a copy of the MEME software please access http://meme-suite.org.

If you use MEME in your research, please cite the following paper:
Timothy L. Bailey and Charles Elkan, "Fitting a mixture model by expectation maximization to discover motifs in biopolymers", *Proceedings of the Second International Conference on Intelligent Systems for Molecular Biology*, pp. 28-36, AAAI Press, Menlo Park, California, 1994. [pdf]

DISCOVERED MOTIFS | MOTIF LOCATIONS | INPUTS & SETTINGS | PROGRAM INFORMATION | RESULTS IN TEXT FORMAT NEW | RESULTS IN XML FORMAT NEW

DISCOVERED MOTIFS

		Logo	E-value	Sites	Width	More	Submit/D
1.	+ -	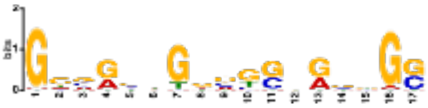	7.4e-003	99691	17		
2.	+ -	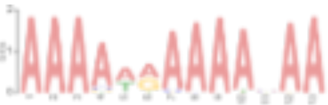	5.7e-001	10361	13		
3.	+ -	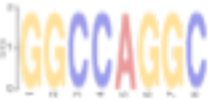	6.5e+000	2226	8		
4.	+ -	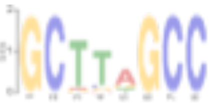	6.5e+000	2990	8		
5.	+ -	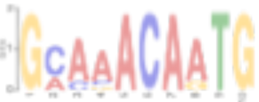	6.7e+000	1993	10		
6.	+ -	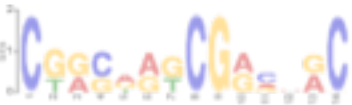	7.0e+000	1158	14		
7.	+ -	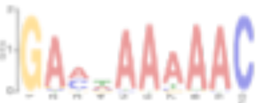	6.3e+000	5669	10		
8.	+ -	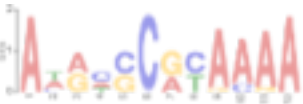	6.2e+000	2535	12		
9.	+ -	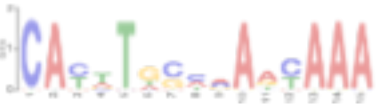	5.5e+000	1547	15		
10.	+ -	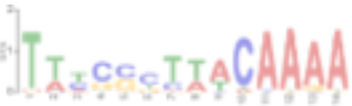	7.0e+000	2633	14		

Stopped because requested number of motifs (10) found.

Motif Locations

Output of motif locations suppressed by -brief option.

Inputs & Settings

Sequences

Role	Source	Alphabet	Sequence Count	Total Size
Primary Sequences	06DiscoMo/Dsim-1k-hi.fa	DNA	21841	54878159
Control Sequences	06DiscoMo/Dsim-1k-lo.fa	DNA	21837	54879163

Background Model

Source: the file '01MarkovModel/dsimM252.1.1.clean.model'

Order: 0

Name	Freq.	Bg.				Bg.	Freq.	Name
Adenine	0.283	0.284	A	~	T	0.284	0.283	Thymine
Cytosine	0.217	0.216	C	~	G	0.216	0.217	Guanine

Other Settings

**Motif Site Distribution**	ANR: Any number of sites per sequence
**Objective Function**	Differential Enrichment mHG
**Starting Point Function**	log likelihood ratio (LLR)
**Site Strand Handling**	Sites may be on either strand
**Maximum Number of Motifs**	10
**Motif E-value Threshold**	no limit
**Minimum Motif Width**	8
**Maximum Motif Width**	50
**Minimum Sites per Motif**	1000
**Maximum Sites per Motif**	109205

Show Advanced Settings

MEME version

5.0.1 (Release date: Thu Jul 26 17:15:19 2018 -0700)

Reference

Timothy L. Bailey and Charles Elkan, "Fitting a mixture model by expectation maximization to discover motifs in biopolymers", *Proceedings of the Second International Conference on Intelligent Systems for Molecular Biology*, pp. 28-36, AAAI Press, Menlo Park, California, 1994.

Command line

```
meme -oc 07MemeDsim-1k -objfun de -neg 06DiscoMo/Dsim-1k-lo.fa -dna -revcomp -mod
anr -nmotifs 10 -minsites 1000 -bfile 01MarkovModel/dsimM252.1.1.clean.model
06DiscoMo/Dsim-1k-hi.fa
```

MEME motif discovery output for *D. simulans*, 5kb windows.

MEME
Multiple Em for Motif Elicitation

For further information on how to interpret these results or to get a copy of the MEME software please access http://meme-suite.org.

If you use MEME in your research, please cite the following paper:
Timothy L. Bailey and Charles Elkan, "Fitting a mixture model by expectation maximization to discover motifs in biopolymers", *Proceedings of the Second International Conference on Intelligent Systems for Molecular Biology*, pp. 28-36, AAAI Press, Menlo Park, California, 1994. [pdf]

Discovered Motifs | Motif Locations | Inputs & Settings | Program Information | Results in Text Format NEW | Results in XML Format NEW

Discovered Motifs

	Logo	E-value	Sites	Width
1.	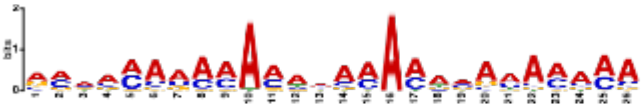	5.3e-023	30563	26
2.	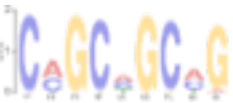	1.7e+000	25180	9
3.	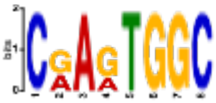	3.8e-002	8197	8
4.	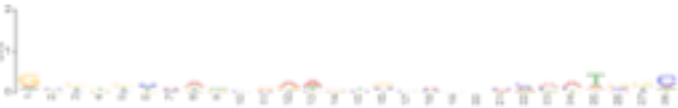	7.0e+000	17429	28
5.	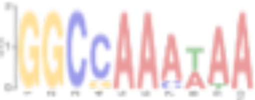	5.6e+000	1762	10
6.	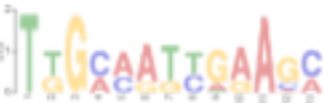	6.5e+000	1458	13
7.	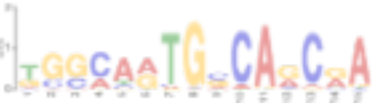	5.5e+000	2587	15
8.	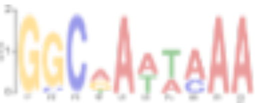	5.6e+000	5950	10
9.	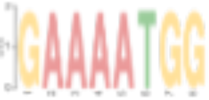	7.0e+000	2339	8
10.	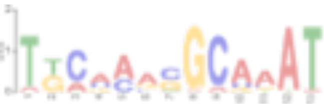	7.0e+000	4655	13

Stopped because requested number of motifs (10) found.

Motif Locations

Output of motif locations suppressed by -brief option.

Inputs & Settings

Sequences

Role	Source	Alphabet	Sequence Count	Total Size
Primary Sequences	06DiscoMo/Dsim-5k-hi.fa	DNA	6806	54890194
Control Sequences	06DiscoMo/Dsim-5k-lo.fa	DNA	6806	54897194

Background Model

Source: the file '01MarkovModel/dsimM252.1.1.clean.model'

Order: 0

Name	Freq.	Bg.				Bg.	Freq.	Name
Adenine	0.283	0.284	A	~	T	0.284	0.283	Thymine
Cytosine	0.217	0.216	C	~	G	0.216	0.217	Guanine

Other Settings

**Motif Site Distribution**	ANR: Any number of sites per sequence
**Objective Function**	Differential Enrichment mHG
**Starting Point Function**	log likelihood ratio (LLR)
**Site Strand Handling**	Sites may be on either strand
**Maximum Number of Motifs**	10
**Motif E-value Threshold**	no limit
**Minimum Motif Width**	8
**Maximum Motif Width**	50
**Minimum Sites per Motif**	1000
**Maximum Sites per Motif**	34030

Show Advanced Settings

MEME version
5.0.1 (Release date: Thu Jul 26 17:15:19 2018 -0700)

Reference
Timothy L. Bailey and Charles Elkan, "Fitting a mixture model by expectation maximization to discover motifs in biopolymers", *Proceedings of the Second International Conference on Intelligent Systems for Molecular Biology*, pp. 28-36, AAAI Press, Menlo Park, California, 1994.

Command line

```
meme -oc 07MemeDsim-5k -objfun de -neg 06DiscoMo/Dsim-5k-lo.fa -dna -revcomp -mod
anr -nmotifs 10 -minsites 1000 -bfile 01MarkovModel/dsimM252.1.1.clean.model
06DiscoMo/Dsim-5k-hi.fa
```

MEME motif discovery output for *D. simulans*, 25kb windows.

MEME

Multiple Em for Motif Elicitation

For further information on how to interpret these results or to get a copy of the MEME software please access http://meme-suite.org.

If you use MEME in your research, please cite the following paper:
Timothy L. Bailey and Charles Elkan, "Fitting a mixture model by expectation maximization to discover motifs in biopolymers", *Proceedings of the Second International Conference on Intelligent Systems for Molecular Biology*, pp. 28-36, AAAI Press, Menlo Park, California, 1994. [pdf]

DISCOVERED MOTIFS | MOTIF LOCATIONS | INPUTS & SETTINGS | PROGRAM INFORMATION | RESULTS IN TEXT FORMAT NEW | RESULTS IN XML FORMAT NEW

DISCOVERED MOTIFS

		Logo	E-value	Sites	Width
1.	+ -	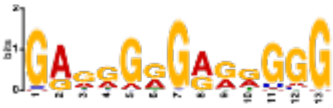	1.5e-019	10516	13
2.	+ -	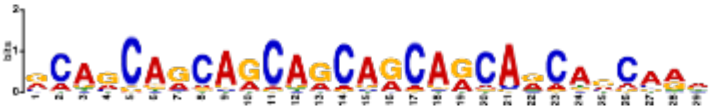	6.3e-013	8161	29
3.	+ -	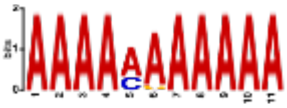	3.1e-006	7841	11
4.	+ -	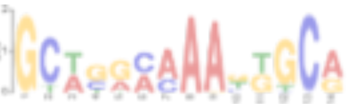	2.6e+000	1024	14
5.	+ -	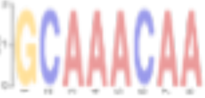	6.1e+000	8082	8
6.	+ -	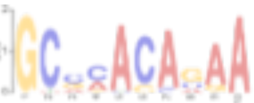	2.4e-001	9282	10
7.	+ -	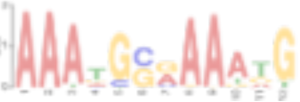	6.7e-002	8275	12
8.	+ -	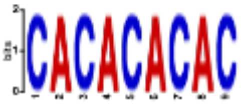	1.4e-016	10560	9
9.	+ -	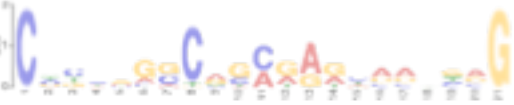	6.7e+000	2504	21
10.	+ -	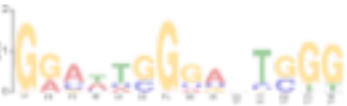	7.0e+000	10559	14

Stopped because requested number of motifs (10) found.

Motif Locations

Output of motif locations suppressed by -brief option.

Inputs & Settings

Sequences

Role	Source	Alphabet	Sequence Count	Total Size
Primary Sequences	06DiscoMo/Dsim-25k-hi.fa	DNA	2112	54888888
Control Sequences	06DiscoMo/Dsim-25k-lo.fa	DNA	2113	54907887

Background Model

Source: the file '01MarkovModel/dsimM252.1.1.clean.model'

Order: 0

Name	Freq.	Bg.				Bg.	Freq.	Name
Adenine	0.283	0.284	A	~	T	0.284	0.283	Thymine
Cytosine	0.217	0.216	C	~	G	0.216	0.217	Guanine

Other Settings

**Motif Site Distribution**	ANR: Any number of sites per sequence
**Objective Function**	Differential Enrichment mHG
**Starting Point Function**	log likelihood ratio (LLR)
**Site Strand Handling**	Sites may be on either strand
**Maximum Number of Motifs**	10
**Motif E-value Threshold**	no limit
**Minimum Motif Width**	8
**Maximum Motif Width**	50
**Minimum Sites per Motif**	1000
**Maximum Sites per Motif**	10560

Show Advanced Settings

MEME version
5.0.1 (Release date: Thu Jul 26 17:15:19 2018 -0700)

Reference
Timothy L. Bailey and Charles Elkan, "Fitting a mixture model by expectation maximization to discover motifs in biopolymers", *Proceedings of the Second International Conference on Intelligent Systems for Molecular Biology*, pp. 28-36, AAAI Press, Menlo Park, California, 1994.

Command line

```
meme -oc 07MemeDsim-25k -objfun de -neg 06DiscoMo/Dsim-25k-lo.fa -dna -revcomp
-mod anr -nmotifs 10 -minsites 1000 -bfile 01MarkovModel/dsimM252.1.1.clean.model
06DiscoMo/Dsim-25k-hi.fa
```

MEME motif discovery output for *D. simulans*, 101kb windows.

MEME
Multiple Em for Motif Elicitation

For further information on how to interpret these results or to get a copy of the MEME software please access http://meme-suite.org.

If you use MEME in your research, please cite the following paper:
Timothy L. Bailey and Charles Elkan, "Fitting a mixture model by expectation maximization to discover motifs in biopolymers", *Proceedings of the Second International Conference on Intelligent Systems for Molecular Biology*, pp. 28-36, AAAI Press, Menlo Park, California, 1994. [pdf]

Discovered Motifs | Motif Locations | Inputs & Settings | Program Information | Results in Text Format NEW | Results in XML Format NEW

Discovered Motifs

	Logo	E-value	Sites	Width	More	Sub
1. + -	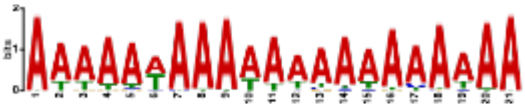	3.9e-003	1514	21	⊥	

Stopped because motif E-value > 1.00e+00.

Motif Locations

Only Motif Sites | Motif Sites+Scanned Sites | All Sequences

Name Location		*p*-value	Motif Location
5.	2L:625001-728000	1.98e-5	+ -
7.	2L:768001-1423000	1.73e-5	+ -
9.	2L:1489001-1751000	2.73e-5	+ -
12.	2L:2173001-2672000	6.71e-4	+ -
21.	2L:3260001-3543000	8.73e-5	+ -
23.	2L:3567001-3569000	1.60e-5	+ -
24.	2L:3619001-3737000	3.05e-4	+ -
27.	2L:4482001-4600000	9.54e-4	+ -
28.	2L:4675001-4796000	3.12e-4	+ -
30.	2L:4805001-4928000	1.39e-4	+ -
31.	2L:5054001-5324000	2.18e-3	+ -
36.	2L:5609001-5668000	3.12e-6	+ -
39.	2L:5956001-6039000	3.55e-5	+ -
40.	2L:6041001-6062000	7.02e-5	+ -

44.	2L:6281001-6282000	2.22e-5	+ -
45.	2L:6636001-6924000	2.48e-5	+ -
49.	2L:6968001-7077000	1.97e-4	+ -
53.	2L:7127001-7419000	8.55e-4	+ -
54.	2L:7521001-7696000	1.07e-4	+ -
59.	2L:8020001-8199000	1.18e-6	+ -
60.	2L:8358001-8390000	5.78e-5	+ -
65.	2L:8521001-8598000	5.34e-5	+ -
66.	2L:8837001-8945000	1.13e-5	+ -
67.	2L:9146001-9942000	4.21e-5	+ -
74.	2L:10400001-10451000	1.57e-5	+ -
79.	2L:10961001-11058000	1.93e-6	+ -
84.	2L:11193001-11408000	2.45e-3	+ -
85.	2L:11515001-11667000	3.63e-5	+ -
86.	2L:11774001-11827000	3.25e-5	+ -
87.	2L:12115001-12238000	2.44e-6	+ -
92.	2L:12615001-12621000	1.15e-6	+ -
93.	2L:12623001-12695000	1.47e-4	+ -
94.	2L:12698001-12745000	3.26e-5	+ -
96.	2L:13321001-13604000	6.76e-5	+ -
102.	2L:13777001-13837000	7.94e-7	+ -
105.	2L:13876001-13877000	4.24e-6	+ -
110.	2L:13926001-14147000	2.14e-4	+ -
115.	2L:14320001-14331000	8.72e-7	+ -
116.	2L:14332001-14333000	4.89e-6	+ -
117.	2L:14453001-14454000	1.32e-6	+ -
118.	2L:14456001-14567000	2.13e-5	+ -
121.	2L:14600001-14638000	7.74e-4	+ -
122.	2L:14642001-14702000	7.94e-7	+ -
123.	2L:14713001-15005000	5.28e-4	+ -
125.	2L:15011001-15161000	7.96e-5	+ -
126.	2L:15266001-15316000	1.26e-3	+ -
129.	2L:15385001-15499000	2.06e-4	+ -
134.	2L:15580001-15583000	5.73e-7	+ -
135.	2L:15585001-15697000	4.24e-4	+ -
136.	2L:15700001-15800000	4.99e-4	+ -

140.	2L:16150001-16213000	4.43e-3	+ -	▌

Inputs & Settings

Sequences

Role	Source	Alphabet	Sequence Count	Total Size
Primary Sequences	06DiscoMo/Dsim-101k-hi_alt.fa	DNA	749	54883253
Control Sequences	06DiscoMo/Dsim-101k-lo_alt.fa	DNA	752	54916251

Background Model

Source: the file '01MarkovModel/dsimM252.1.1.clean.model'

Order: 0

Name	Freq.	Bg.				Bg.	Freq.	Name
Adenine	0.283	0.284	A	~	T	0.284	0.283	Thymine
Cytosine	0.217	0.216	C	~	G	0.216	0.217	Guanine

Other Settings

**Motif Site Distribution**	ANR: Any number of sites per sequence
**Objective Function**	Differential Enrichment mHG
**Starting Point Function**	log likelihood ratio (LLR)
**Site Strand Handling**	Sites may be on either strand
**Maximum Number of Motifs**	10
**Motif E-value Threshold**	1
**Minimum Motif Width**	8
**Maximum Motif Width**	50
**Minimum Sites per Motif**	1000
**Maximum Sites per Motif**	3745

Show Advanced Settings

MEME version

5.0.1 (Release date: Thu Jul 26 17:15:19 2018 -0700)

Reference

Timothy L. Bailey and Charles Elkan, "Fitting a mixture model by expectation maximization to discover motifs in biopolymers", *Proceedings of the Second International Conference on Intelligent Systems for Molecular Biology*, pp. 28-36, AAAI Press, Menlo Park, California, 1994.

Command line

```
meme -oc 07MemeDsim-101k -objfun de -neg 06DiscoMo/Dsim-101k-lo_alt.fa -dna
-revcomp -mod anr -nmotifs 10 -evt 1 -minsites 1000 -bfile
01MarkovModel/dsimM252.1.1.clean.model 06DiscoMo/Dsim-101k-hi_alt.fa
```

MEME motif discovery output for *D. simulans*, 501kb windows.

MEME

Multiple Em for Motif Elicitation

For further information on how to interpret these results or to get a copy of the MEME software please access http://meme-suite.org.

If you use MEME in your research, please cite the following paper:
Timothy L. Bailey and Charles Elkan, "Fitting a mixture model by expectation maximization to discover motifs in biopolymers", *Proceedings of the Second International Conference on Intelligent Systems for Molecular Biology*, pp. 28-36, AAAI Press, Menlo Park, California, 1994. [pdf]

DISCOVERED MOTIFS | MOTIF LOCATIONS | INPUTS & SETTINGS | PROGRAM INFORMATION | RESULTS IN TEXT FORMAT NEW | RESULTS IN XML FORMAT NEW

DISCOVERED MOTIFS

		Logo	E-valu
1.	+ -	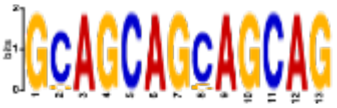	5.1e-0
2.	+ -	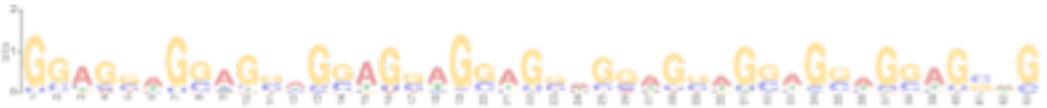	2.7e-0

Stopped because motif E-value > 1.00e+00.

MOTIF LOCATIONS

Only Motif Sites | Motif Sites+Scanned Sites | All Sequences

	Location	*p*-value	Motif Location
1.	2L:617001-1617001	1.37e-16	+ -
2.	2L:1617001-1875000	8.80e-8	+ -
3.	2L:2047001-2654000	7.46e-13	+ -
5.	2L:2784001-2795000	3.22e-9	+ -
6.	2L:2816001-2830000	5.70e-9	+ -
7.	2L:3089001-3649000	4.65e-10	+ -
8.	2L:4613001-4763000	5.02e-8	+ -
11.	2L:4858001-5449000	1.47e-9	+ -
12.	2L:6625001-6681000	1.33e-12	+ -
13.	2L:6692001-7628000	4.75e-9	+ -
15.	2L:7689001-7876000	9.28e-14	+ -
17.	2L:7887001-7908000	9.65e-14	+ -

22.	2L:8089001-8200000	1.45e-9
24.	2L:8255001-8274000	4.59e-8
25.	2L:8302001-8311000	5.70e-5
27.	2L:9228001-9904000	5.14e-12
28.	2L:11030001-11627000	2.42e-10
34.	2L:13266001-14163000	1.71e-10
37.	2L:14467001-15467001	1.96e-12
38.	2L:15467001-15673000	2.18e-8
40.	2L:15679001-15684000	9.07e-9
45.	2L:17652001-18652001	4.60e-11
46.	2L:18652001-19652001	1.58e-11
47.	2L:19652001-20038000	6.78e-13
48.	2R:1360001-2274000	1.67e-14
49.	2R:2297001-2316000	3.62e-17
51.	2R:3503001-3509000	1.00e-11
52.	2R:3638001-3851000	1.32e-9
54.	2R:5243001-5831000	3.90e-11
56.	2R:6896001-7128000	2.05e-9
57.	2R:7619001-8059000	3.50e-9
60.	2R:8195001-8693000	3.36e-9
61.	2R:9637001-10632000	6.24e-16
63.	2R:11554001-11566000	4.72e-6
67.	2R:11584001-11627000	4.97e-9
68.	2R:11707001-12005000	6.31e-12
69.	2R:12027001-12083000	2.41e-8
71.	2R:12325001-12531000	1.25e-10
72.	2R:12626001-12786000	1.20e-10
74.	2R:13021001-13147000	3.57e-11
75.	2R:13232001-14232001	2.49e-10
76.	2R:14232001-15232001	1.28e-13
77.	2R:15232001-15664000	1.09e-11
78.	2R:15665001-15925000	1.06e-10
79.	2R:16140001-16194000	2.75e-9
82.	2R:16550001-16656000	4.55e-12
86.	2R:16706001-17073000	3.62e-11
87.	2R:17174001-17243000	4.42e-12

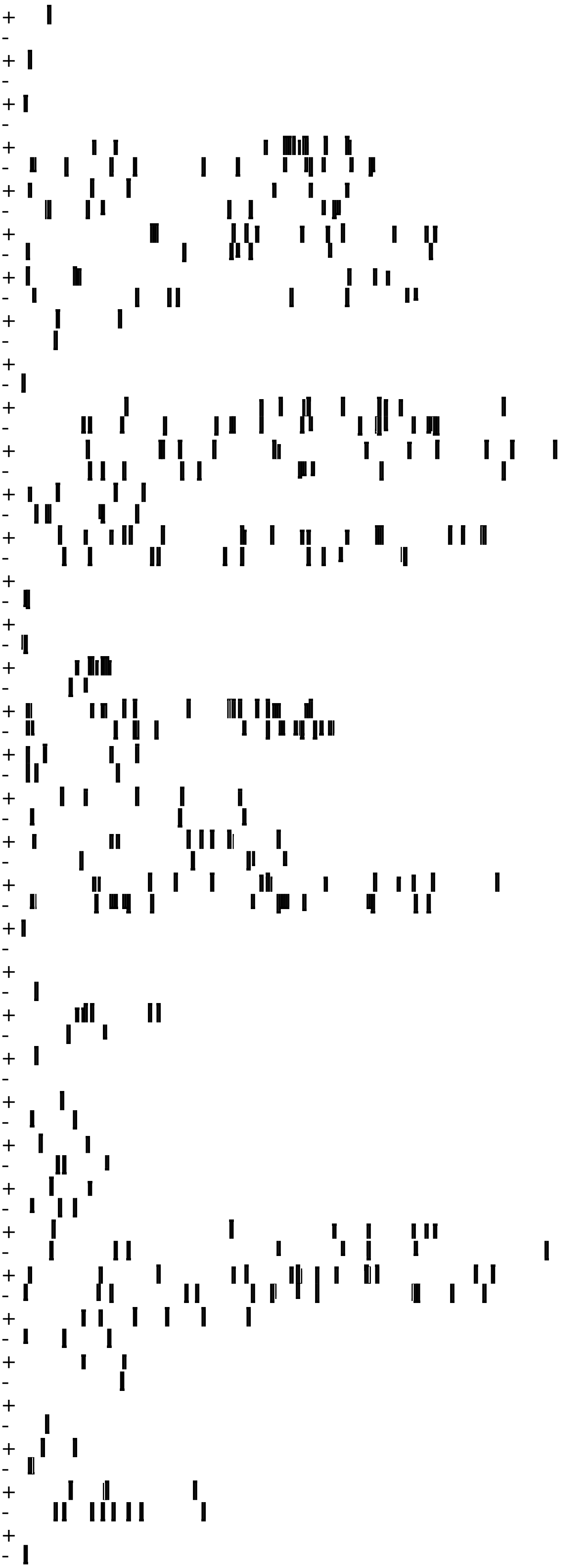

88.	2R:17275001-18275001	5.13e-14	+ -
89.	2R:18275001-18539000	5.93e-12	+ -
90.	3L:179001-311000	4.48e-12	+ -
91.	3L:362001-1362001	4.04e-10	+ -
92.	3L:1362001-2067000	2.35e-11	+ -
95.	3L:2567001-2724000	8.58e-11	+ -
96.	3L:3334001-4334001	5.47e-11	+ -
97.	3L:4334001-4398000	5.31e-6	+ -
98.	3L:4406001-4698000	9.04e-18	+ -
100.	3L:4738001-5446000	4.67e-12	+ -

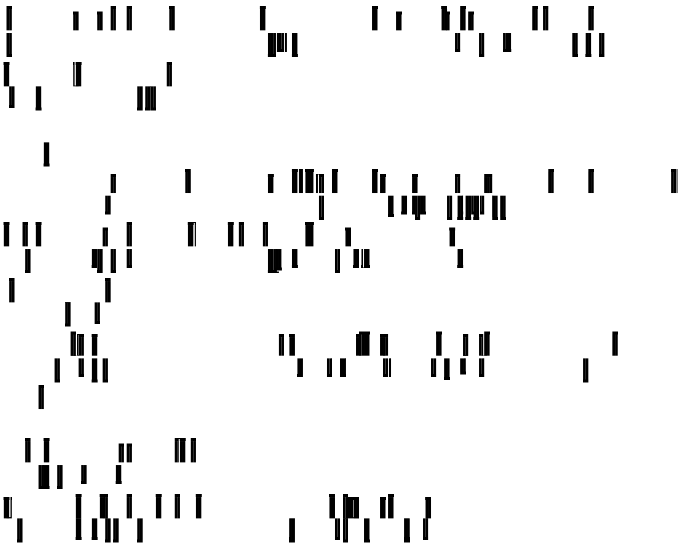

Inputs & Settings

Sequences

Role	Source	Alphabet	Sequence Count	Total Size
Primary Sequences	06DiscoMo/Dsim-501k-hi_alt.fa	DNA	287	54862732
Control Sequences	06DiscoMo/Dsim-501k-lo_alt.fa	DNA	293	54937728

Background Model

Source: the file '01MarkovModel/dsimM252.1.1.clean.model'

Order: 0

Name	Freq.	Bg.				Bg.	Freq.	Name
Adenine	0.282	0.284	A	~	T	0.284	0.282	Thymine
Cytosine	0.218	0.216	C	~	G	0.216	0.218	Guanine

Other Settings

**Motif Site Distribution**	ANR: Any number of sites per sequence
**Objective Function**	Differential Enrichment mHG
**Starting Point Function**	log likelihood ratio (LLR)
**Site Strand Handling**	Sites may be on either strand
**Maximum Number of Motifs**	10
**Motif E-value Threshold**	1
**Minimum Motif Width**	8
**Maximum Motif Width**	50
**Minimum Sites per Motif**	1000
**Maximum Sites per Motif**	1435

Show Advanced Settings

MEME version

5.0.1 (Release date: Thu Jul 26 17:15:19 2018 -0700)

Reference

Timothy L. Bailey and Charles Elkan, "Fitting a mixture model by expectation maximization to discover motifs in biopolymers", *Proceedings of the Second International Conference on Intelligent Systems for Molecular Biology*, pp. 28-36, AAAI Press, Menlo Park, California, 1994.

Command line

```
meme -oc 07MemeDsim-501k -objfun de -neg 06DiscoMo/Dsim-501k-lo_alt.fa -dna
-revcomp -mod anr -nmotifs 10 -evt 1 -minsites 1000 -bfile
01MarkovModel/dsimM252.1.1.clean.model 06DiscoMo/Dsim-501k-hi_alt.fa
```

MEME motif discovery output for *D. simulans*, 2501kb windows.

MEME

Multiple Em for Motif Elicitation

For further information on how to interpret these results or to get a copy of the MEME software please access http://meme-suite.org.

If you use MEME in your research, please cite the following paper:
Timothy L. Bailey and Charles Elkan, "Fitting a mixture model by expectation maximization to discover motifs in biopolymers", *Proceedings of the Second International Conference on Intelligent Systems for Molecular Biology*, pp. 28-36, AAAI Press, Menlo Park, California, 1994. [pdf]

DISCOVERED MOTIFS | MOTIF LOCATIONS | INPUTS & SETTINGS | PROGRAM INFORMATION | RESULTS IN TEXT FORMAT NEW | RESULTS IN XML FORMAT NEW

DISCOVERED MOTIFS

		Logo	E-value	Sites	Width	Mo
1.	+ -	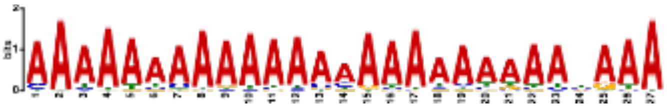	2.7e-002	1294	27	
2.	+ -	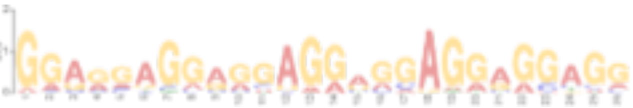	4.3e-001	1167	26	
3.	+ -	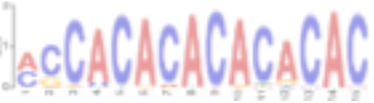	8.4e-001	1215	15	
4.	+ -	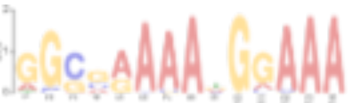	1.4e-001	1058	14	

Stopped because motif E-value > 1.00e+00.

MOTIF LOCATIONS

Only Motif Sites   Motif Sites+Scanned Sites   All Sequences

	Name Location	*p*-value	Motif Location
4.	2L:1140001-1640001	4.78e-12	+ -
5.	2L:1640001-2140001	8.80e-10	+ -
6.	2L:2140001-2640001	1.16e-11	+ -
7.	2L:2640001-2983000	7.07e-11	+ -
10.	2L:3354001-3443000	9.02e-10	+ -
13.	2L:4090001-4198000	4.81e-9	+ -
16.	2L:5916001-5985000	1.56e-8	+ -
18.	2L:6029001-6082000	4.55e-12	+ -

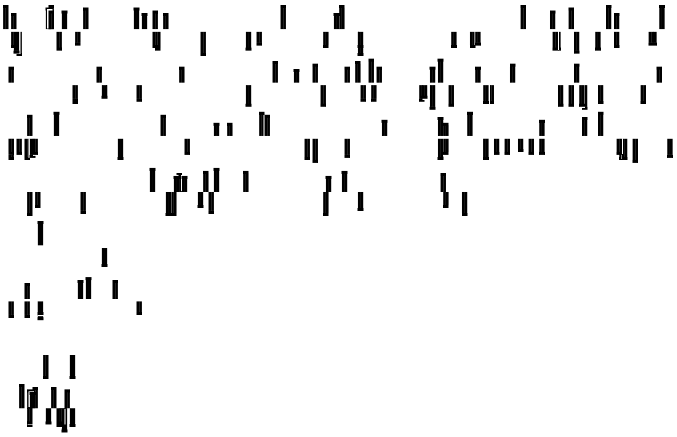

20.	2L:6096001-6190000	3.46e-10	+ -
23.	2L:6228001-6487000	3.72e-13	+ -
24.	2L:6816001-6933000	1.08e-11	+ -
26.	2L:6946001-7446001	1.22e-13	+ -
27.	2L:7446001-7570000	2.71e-9	+ -
28.	2L:7594001-8094001	1.76e-15	+ -
29.	2L:8094001-8594001	1.44e-15	+ -
30.	2L:8594001-8909000	9.12e-11	+ -
31.	2L:8973001-9417000	2.59e-14	+ -
32.	2L:10031001-10531001	1.91e-14	+ -
33.	2L:10531001-10723000	3.76e-16	+ -
34.	2L:13679001-14179001	5.02e-11	+ -
35.	2L:14179001-14679001	9.47e-11	+ -
36.	2L:14679001-15179001	4.52e-12	+ -
37.	2L:15179001-15403000	1.12e-7	+ -
39.	2L:15452001-15929000	5.81e-14	+ -
40.	2L:15933001-15934000	1.04e-7	+ -
41.	2L:15935001-15943000	6.52e-13	+ -
43.	2L:16038001-16076000	2.09e-8	+ -
44.	2L:16079001-16124000	1.28e-6	+ -
45.	2L:16931001-17022000	3.19e-11	+ -
46.	2L:17032001-17044000	2.12e-9	+ -
48.	2L:17138001-17139000	5.14e-8	+ -
49.	2L:17140001-17640001	1.76e-7	+ -
50.	2L:17640001-18140001	8.13e-12	+ -
51.	2L:18140001-18640001	2.09e-13	+ -
52.	2L:18640001-19140001	8.22e-13	+ -
53.	2L:19140001-19445000	2.58e-12	+ -
55.	2R:2476001-2491000	8.44e-6	+ -
59.	2R:2548001-2598000	5.89e-17	+ -
61.	2R:2651001-2720000	4.94e-10	+ -
63.	2R:4526001-4781000	4.02e-13	+ -
64.	2R:7735001-7737000	5.94e-6	+ -
65.	2R:7738001-7998000	1.44e-10	+ -
67.	2R:8838001-8864000	1.83e-11	+ -
69.	2R:8882001-9055000	4.38e-13	+ -

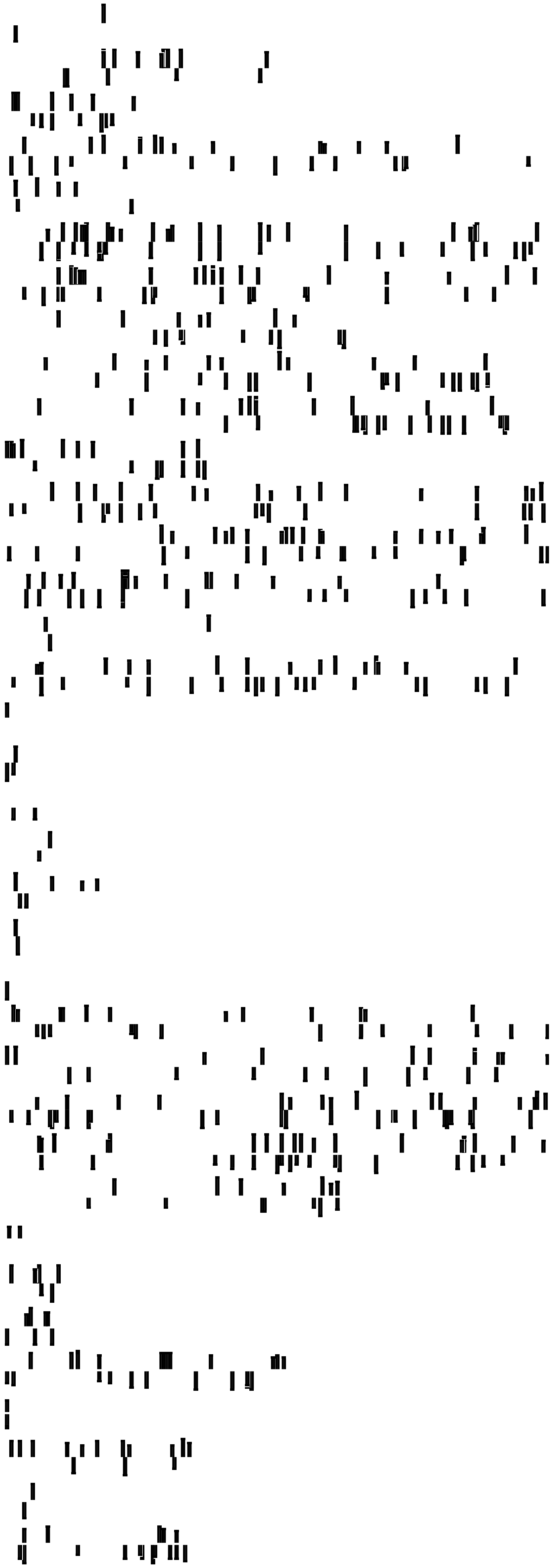

71.	2R:9064001-9073000	4.55e-8
72.	2R:9165001-9216000	9.63e-19
73.	2R:9314001-9320000	7.61e-9
74.	2R:9321001-9675000	8.77e-12
76.	2R:10516001-11016001	5.92e-11
77.	2R:11016001-11516001	2.97e-14
81.	2R:11611001-11780000	5.81e-10
83.	2R:12062001-12067000	3.95e-7
84.	2R:12069001-12169000	3.07e-13
85.	2R:12171001-12213000	1.46e-10
86.	2R:12223001-12723001	1.55e-13
87.	2R:12723001-13223001	2.66e-12
88.	2R:13223001-13723001	6.40e-12
89.	2R:13723001-14223001	6.37e-12
90.	2R:14223001-14723001	3.31e-13
91.	2R:14723001-15223001	4.83e-12
92.	2R:15223001-15723001	1.20e-15
93.	2R:15723001-16223001	5.24e-13
94.	2R:16223001-16723001	2.64e-12
95.	2R:16723001-17223001	8.78e-13
96.	2R:17223001-17723001	4.79e-13
97.	2R:17723001-18223001	2.82e-12
98.	2R:18223001-18723001	1.67e-14
99.	2R:18723001-18960000	9.05e-7
100.	3L:1-500001	1.70e-18

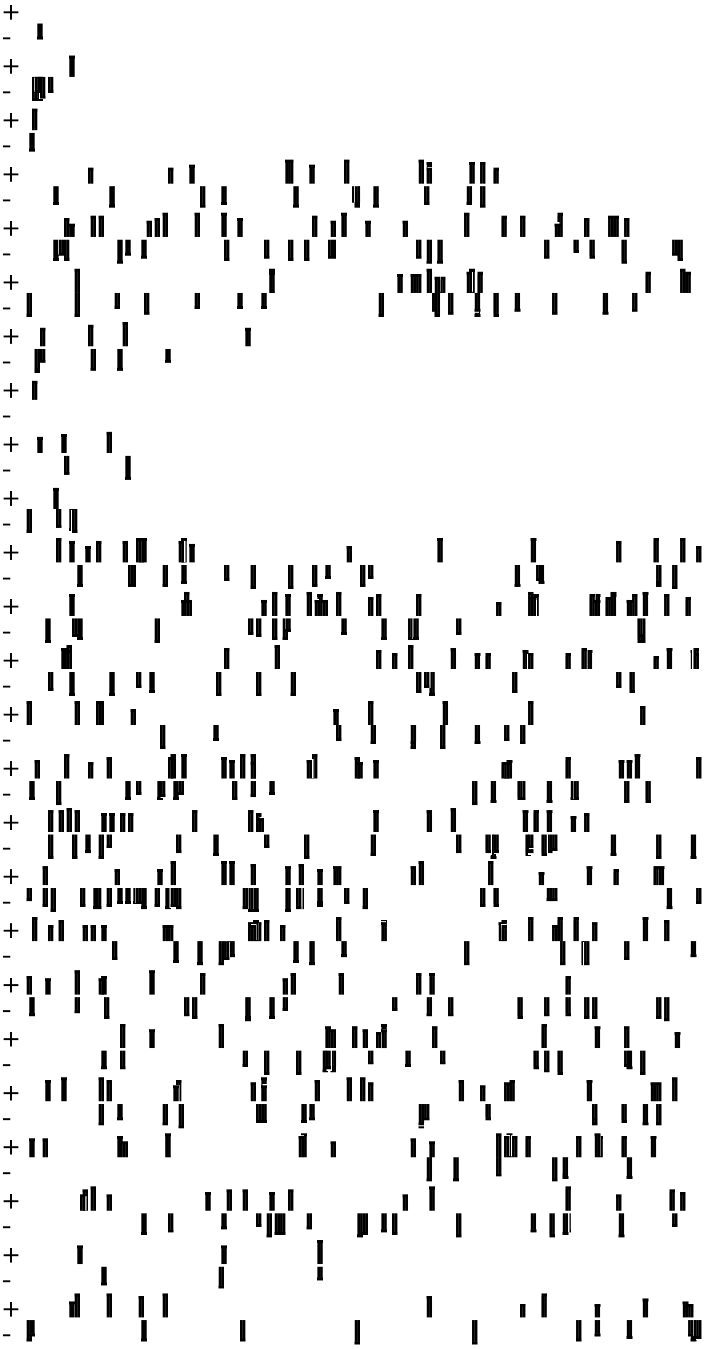

Inputs & Settings

Sequences

Role	Source	Alphabet	Sequence Count	Total Size
Primary Sequences	06DiscoMo/Dsim-2501k-hi_alt.fa	DNA	262	54840822
Control Sequences	06DiscoMo/Dsim-2501k-lo_alt.fa	DNA	264	54959821

Background Model

Source: the file '01MarkovModel/dsimM252.1.1.clean.model'

Order: 0

Name	Freq.	Bg.				Bg.	Freq.	Name
Adenine	0.282	0.284	A	~	T	0.284	0.282	Thymine
Cytosine	0.218	0.216	C	~	G	0.216	0.218	Guanine

Other Settings

**Motif Site Distribution**	ANR: Any number of sites per sequence
**Objective Function**	Differential Enrichment mHG
**Starting Point Function**	log likelihood ratio (LLR)
**Site Strand Handling**	Sites may be on either strand
**Maximum Number of Motifs**	10
**Motif E-value Threshold**	1
**Minimum Motif Width**	8
**Maximum Motif Width**	50
**Minimum Sites per Motif**	1000
**Maximum Sites per Motif**	1310

Show Advanced Settings

MEME version

5.0.1 (Release date: Thu Jul 26 17:15:19 2018 -0700)

Reference

Timothy L. Bailey and Charles Elkan, "Fitting a mixture model by expectation maximization to discover motifs in biopolymers", *Proceedings of the Second International Conference on Intelligent Systems for Molecular Biology*, pp. 28-36, AAAI Press, Menlo Park, California, 1994.

Command line

```
meme -oc 07MemeDsim-2501k -objfun de -neg 06DiscoMo/Dsim-2501k-lo_alt.fa -dna
-revcomp -mod anr -nmotifs 10 -evt 1 -minsites 1000 -bfile
01MarkovModel/dsimM252.1.1.clean.model 06DiscoMo/Dsim-2501k-hi_alt.fa
```
