# Supplementary material for: DNA Motifs Are Not General Predictors of Recombination in Two Drosophila Sister Species
Source: Genome Biol Evol. 2019 Apr 15;11(4):1345–57. doi: 10.1093/gbe/evz082 (PMC6490297; doi:10.1093/gbe/evz082)
Supplement: Supplementary Data [file evz082_supp.zip › SI.4.pdf]

Howie et al. Supplementary Information IV [S4]

TomTom contrast of motifs from Adrian et al. (2016) to our set of 5 consensus motifs.

For further information on how to interpret these results or to get a copy of the MEME software please access http://meme.nbcr.net.

If you use TOMTOM in your research, please cite the following paper:
Shobhit Gupta, JA Stamatoyannopolous, Timothy Bailey and William Stafford Noble, "Quantifying similarity between motifs", *Genome Biology*, **8**(2):R24, 2007. [full text]

QUERY MOTIFS | TARGET DATABASES | MATCHES | SETTINGS | PROGRAM INFORMATION | RESULTS IN TSV FORMAT NEW | RESULTS IN XML FORMAT

QUERY MOTIFS

Next Top

Database	ID	Alt. ID	Preview	Matches	List
adrian-motifs.txt	M1		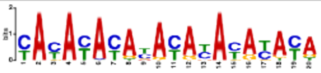	3	C3, C4, C1
adrian-motifs.txt	M10		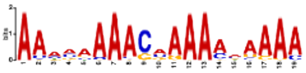	1	C1
adrian-motifs.txt	M11		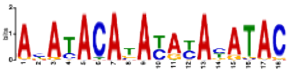	3	C4, C3, C1
adrian-motifs.txt	M12		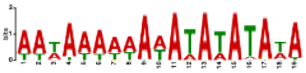	2	C4, C1
adrian-motifs.txt	M13		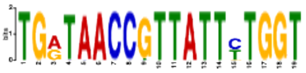	0	
adrian-motifs.txt	M14		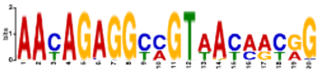	0	
adrian-motifs.txt	M15		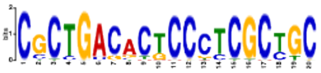	2	C5, C2
adrian-motifs.txt	M16		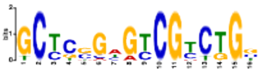	0	
adrian-motifs.txt	M17		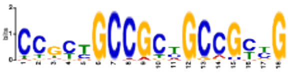	2	C2, C5
adrian-motifs.txt	M18		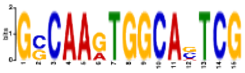	0	
adrian-motifs.txt	M19		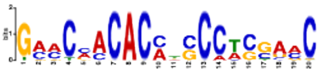	2	C5, C3
adrian-motifs.txt	M2		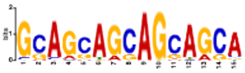	1	C2
adrian-motifs.txt	M20		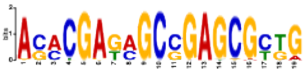	0	
adrian-motifs.txt	M3		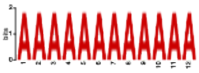	1	C1
adrian-motifs.txt	M4		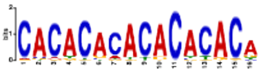	1	C3
adrian-motifs.txt	M5		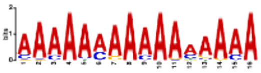	1	C1
adrian-motifs.txt	M6		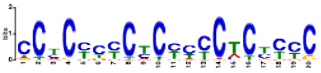	1	C5
adrian-motifs.txt	M7		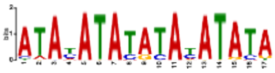	1	C4
adrian-motifs.txt	M8		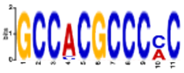	2	C5, C3

Database	ID	Alt. ID	Preview	Matches	List
adrian-motifs.txt	M9		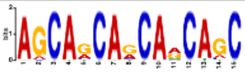	1	C2

Target Databases

Previous Next Top

Database	Used	Matched
consensus-motifs2.txt	5	5

Matches to M1

Previous Next Top

Summary

**Name**	C3
**Database**	consensus-motifs2.txt
***p*-value**	2.66e-05
***E*-value**	1.33e-04
***q*-value**	2.66e-04
**Overlap**	8
**Offset**	0
**Orientation**	Normal

Show logo download options

Optimal Alignment

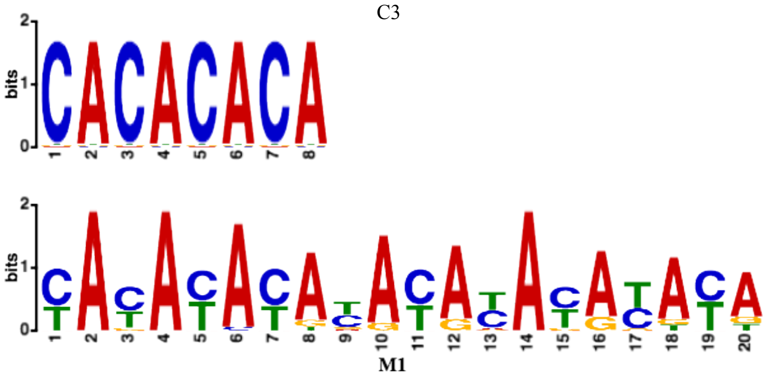

Summary

**Name**	C4
**Database**	consensus-motifs2.txt
***p*-value**	1.26e-03
***E*-value**	6.29e-03
***q*-value**	4.19e-03
**Overlap**	10
**Offset**	0
**Orientation**	Normal

Show logo download options

Optimal Alignment

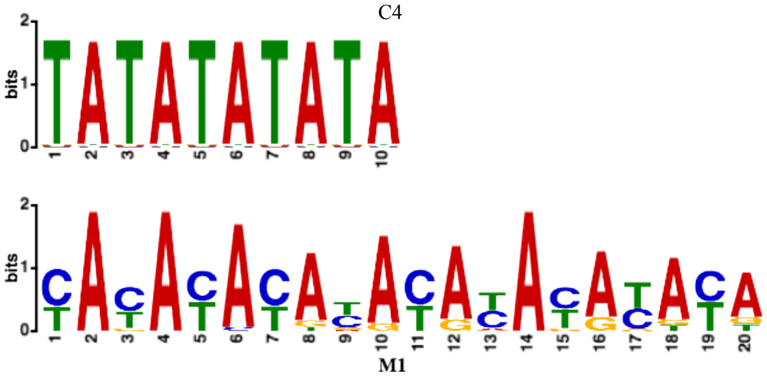

Summary

**Name**	C1
**Database**	consensus-motifs2.txt
***p*-value**	5.60e-02
***E*-value**	2.80e-01
***q*-value**	1.40e-01
**Overlap**	11
**Offset**	-3
**Orientation**	Normal

Show logo download options

Optimal Alignment

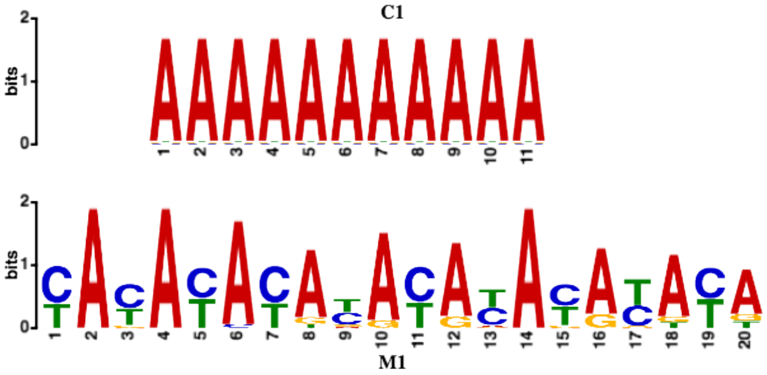

Matches to M2

Previous Next Top

Summary

Optimal Alignment

**Name**	C2
**Database**	consensus-motifs2.txt
***p*-value**	9.23e-08
***E*-value**	4.61e-07
***q*-value**	9.23e-07
**Overlap**	12
**Offset**	0
**Orientation**	Normal

Show logo download options

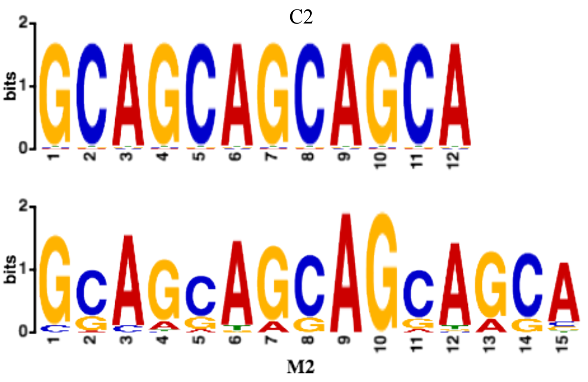

Matches to M3

Previous Next Top

**Summary**	
**Name**	C1
**Database**	consensus-motifs2.txt
***p*-value**	6.09e-06
***E*-value**	3.05e-05
***q*-value**	6.09e-05
**Overlap**	11
**Offset**	-1
**Orientation**	Normal

Show logo download options

Optimal Alignment

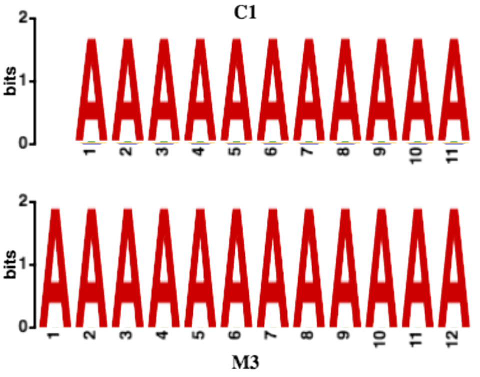

Matches to M4

Previous Next Top

**Summary**	
**Name**	C3
**Database**	consensus-motifs2.txt
***p*-value**	5.32e-05
***E*-value**	2.66e-04
***q*-value**	5.32e-04
**Overlap**	8
**Offset**	-6
**Orientation**	Normal

Show logo download options

Optimal Alignment

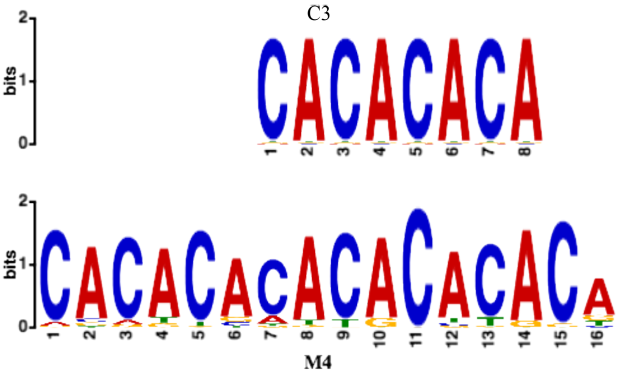

Matches to M5

Previous Next Top

**Summary**	
**Name**	C1
**Database**	consensus-motifs2.txt
***p*-value**	3.05e-06
***E*-value**	1.52e-05
***q*-value**	3.05e-05
**Overlap**	11
**Offset**	0
**Orientation**	Normal

Show logo download options

Optimal Alignment

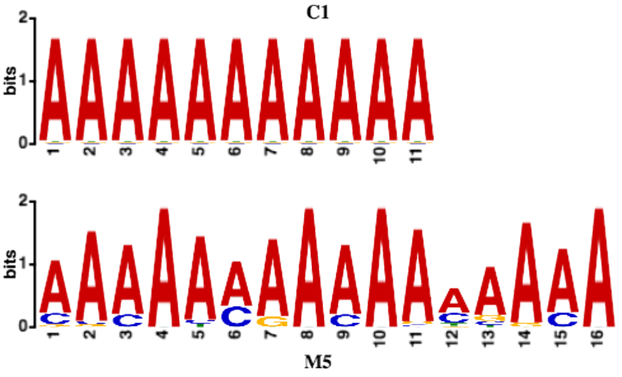

Matches to M6

Previous Next Top

Summary

**Name**	C5
**Database**	consensus-motifs2.txt
***p*-value**	2.74e-04
***E*-value**	1.37e-03
***q*-value**	2.74e-03
**Overlap**	8
**Offset**	0
**Orientation**	Reverse Complement

Show logo download options

Optimal Alignment

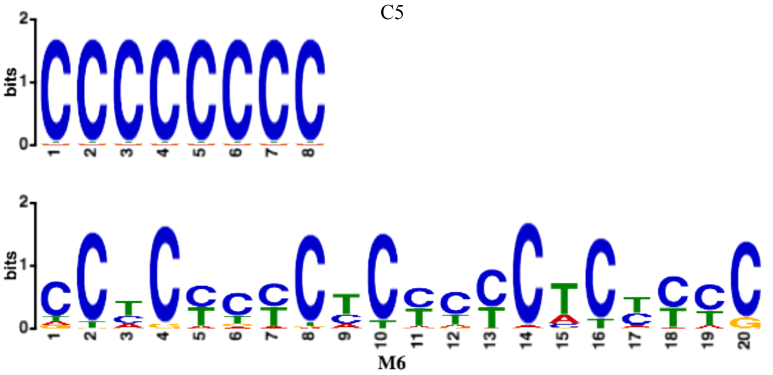

Matches to M7

Previous Next Top

Summary

**Name**	C4
**Database**	consensus-motifs2.txt
***p*-value**	2.06e-05
***E*-value**	1.03e-04
***q*-value**	1.03e-04
**Overlap**	10
**Offset**	-5
**Orientation**	Normal

Show logo download options

Optimal Alignment

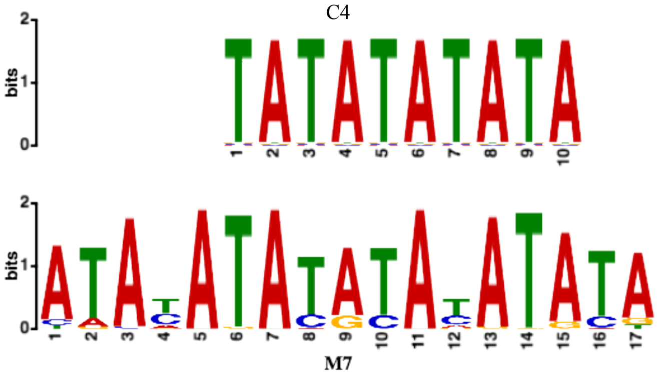

Matches to M8

Previous Next Top

Summary

**Name**	C5
**Database**	consensus-motifs2.txt
***p*-value**	2.22e-03
***E*-value**	1.11e-02
***q*-value**	2.22e-02
**Overlap**	8
**Offset**	-1
**Orientation**	Reverse Complement

Show logo download options

Optimal Alignment

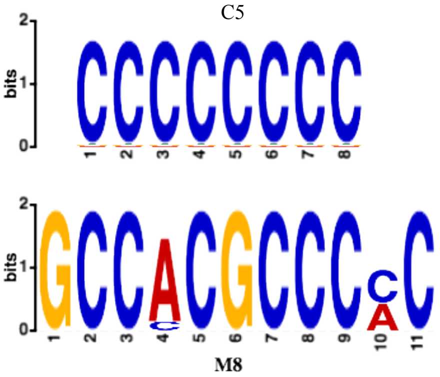

Summary

**Name**	C3
**Database**	consensus-motifs2.txt
***p*-value**	2.49e-02
***E*-value**	1.25e-01
***q*-value**	1.25e-01
**Overlap**	8
**Offset**	-2
**Orientation**	Normal

Show logo download options

Optimal Alignment

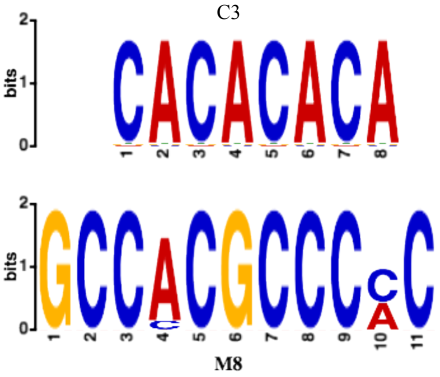

Matches to M9

Previous Next Top

Summary

**Name**	C2
**Database**	consensus-motifs2.txt
***p*-value**	2.26e-07
***E*-value**	1.13e-06
***q*-value**	2.26e-06
**Overlap**	12
**Offset**	-1
**Orientation**	Normal

Show logo download options

Optimal Alignment

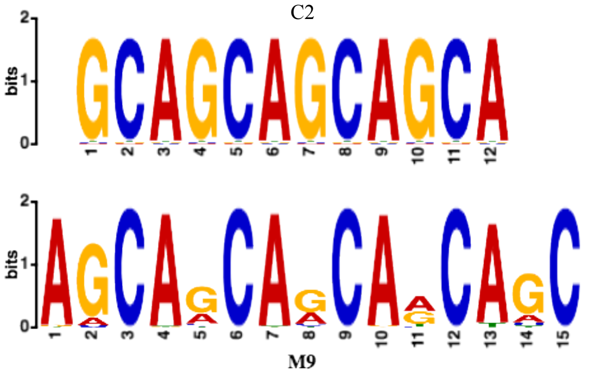

Matches to M10

Previous Next Top

Summary

**Name**	C1
**Database**	consensus-motifs2.txt
***p*-value**	2.78e-04
***E*-value**	1.39e-03
***q*-value**	2.78e-03
**Overlap**	11
**Offset**	-5
**Orientation**	Normal

Show logo download options

Optimal Alignment

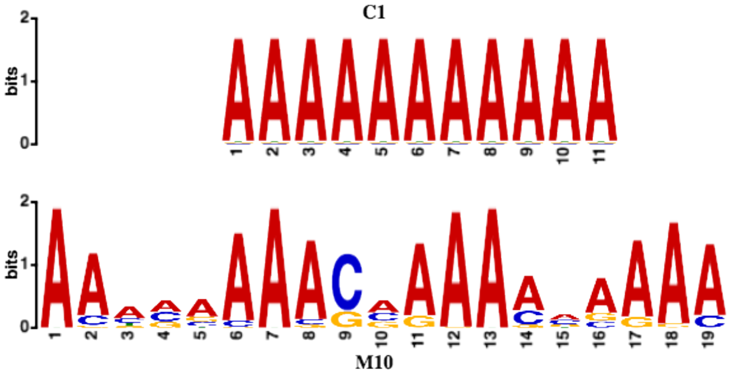

Matches to M11

Previous Next Top

Summary

**Name**	C4
**Database**	consensus-motifs2.txt
***p*-value**	1.01e-03
***E*-value**	5.07e-03
***q*-value**	5.07e-03
**Overlap**	10
**Offset**	-7
**Orientation**	Normal

Show logo download options

Optimal Alignment

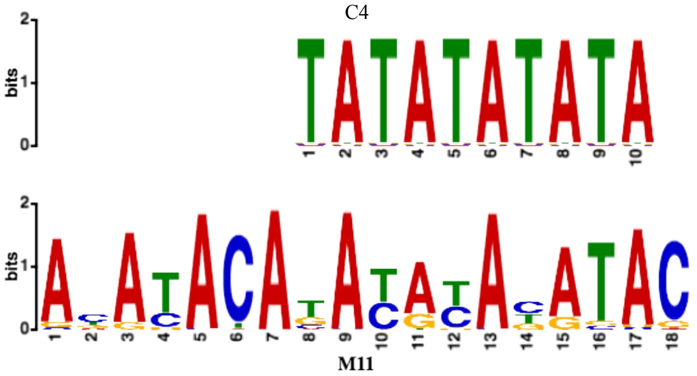

Summary

**Name**	C3
**Database**	consensus-motifs2.txt
***p*-value**	1.01e-02
***E*-value**	5.04e-02
***q*-value**	3.36e-02
**Overlap**	8
**Offset**	-1
**Orientation**	Normal

Show logo download options

Optimal Alignment

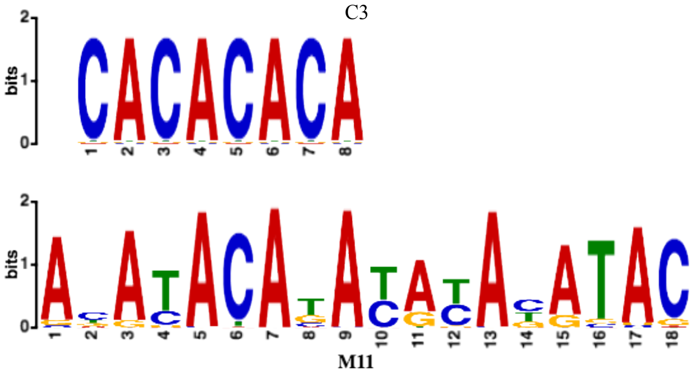

Summary

Optimal Alignment

**Name**	C1
**Database**	consensus-motifs2.txt
***p*-value**	1.09e-01
***E*-value**	5.43e-01
***q*-value**	2.71e-01
**Overlap**	11
**Offset**	-2
**Orientation**	Normal

Show logo download options

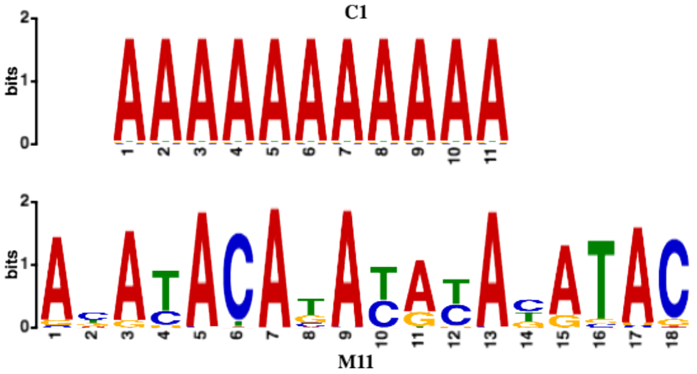

Matches to M12

Previous Next Top

Summary | **Optimal Alignment**

**Name**	C4
**Database**	consensus-motifs2.txt
***p*-value**	1.17e-04
***E*-value**	5.86e-04
***q*-value**	5.86e-04
**Overlap**	10
**Offset**	-9
**Orientation**	Normal

Show logo download options

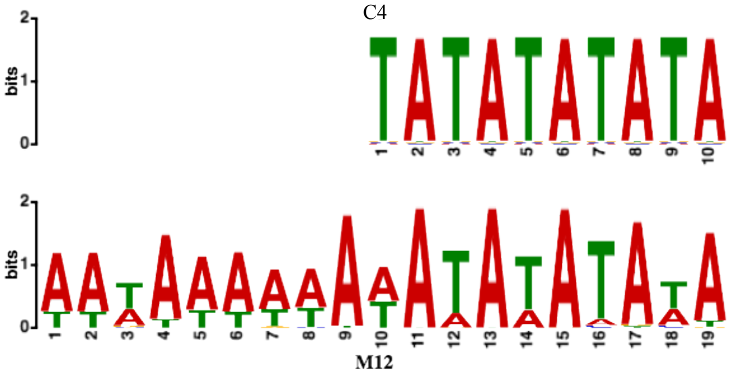

Summary | **Optimal Alignment**

**Name**	C1
**Database**	consensus-motifs2.txt
***p*-value**	9.53e-03
***E*-value**	4.77e-02
***q*-value**	3.18e-02
**Overlap**	11
**Offset**	0
**Orientation**	Normal

Show logo download options

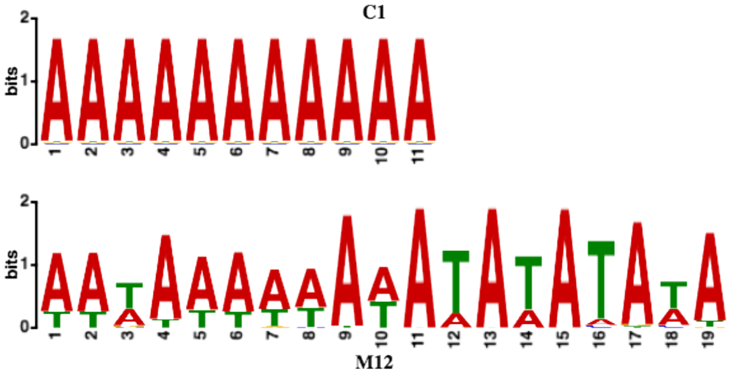

Matches to M15

Previous Next Top

Summary | **Optimal Alignment**

**Name**	C5
**Database**	consensus-motifs2.txt
***p*-value**	5.43e-02
***E*-value**	2.71e-01
***q*-value**	4.25e-01
**Overlap**	8
**Offset**	-10
**Orientation**	Reverse Complement

Show logo download options

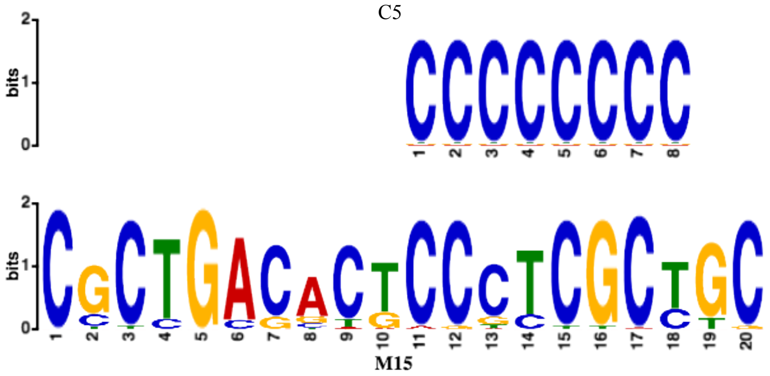

Summary | **Optimal Alignment**

**Name**	C2
**Database**	consensus-motifs2.txt
***p*-value**	8.50e-02
***E*-value**	4.25e-01
***q*-value**	4.25e-01
**Overlap**	12
**Offset**	0
**Orientation**	Reverse Complement

Show logo download options

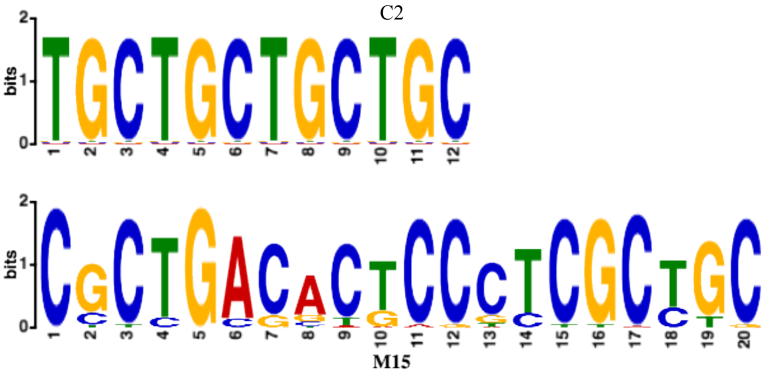

Matches to M17

Previous Next Top

Summary | **Optimal Alignment**

**Name**	C2
**Database**	consensus-motifs2.txt
***p*-value**	1.26e-04
***E*-value**	6.29e-04
***q*-value**	1.26e-03
**Overlap**	12
**Offset**	-4
**Orientation**	Reverse Complement

Show logo download options

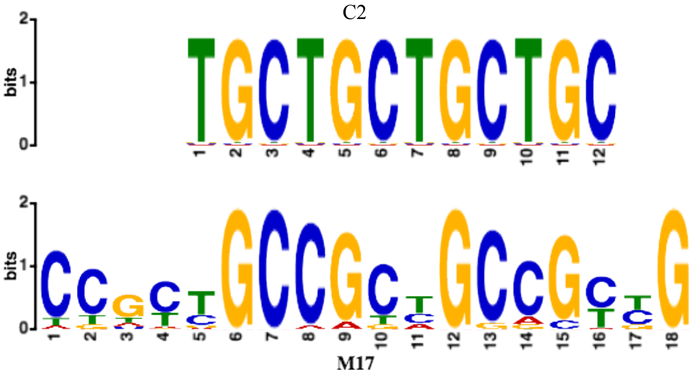

Summary | **Optimal Alignment**

**Name**	C5
**Database**	consensus-motifs2.txt
***p*-value**	4.66e-02
***E*-value**	2.33e-01
***q*-value**	1.55e-01
**Overlap**	8
**Offset**	-6
**Orientation**	Reverse Complement

Show logo download options

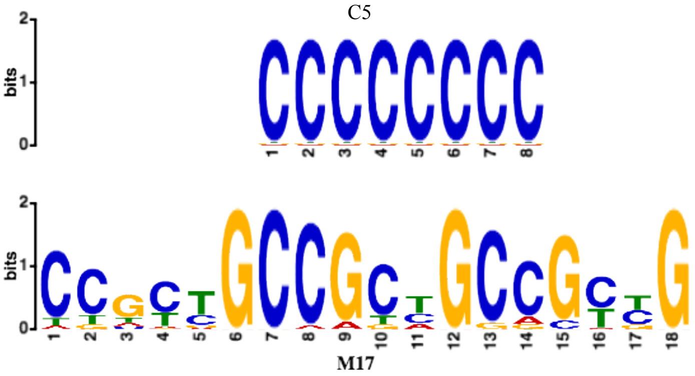

Matches to M19

Previous Next Top

Summary | **Optimal Alignment**

**Name**	C5
**Database**	consensus-motifs2.txt
***p*-value**	1.29e-02
***E*-value**	6.47e-02
***q*-value**	1.29e-01
**Overlap**	8
**Offset**	-6
**Orientation**	Reverse Complement

Show logo download options

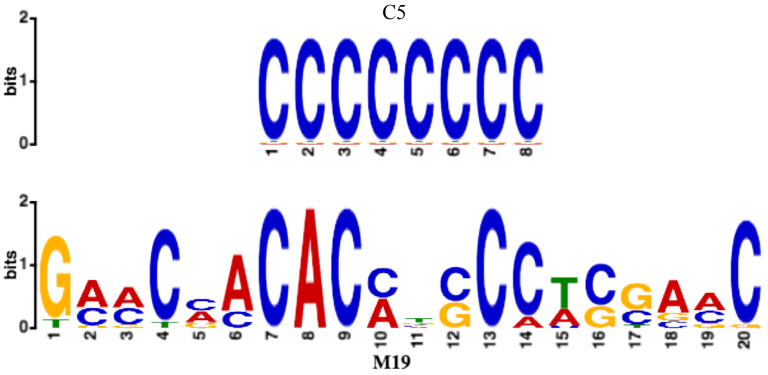

Summary | **Optimal Alignment**

**Name**	C3
**Database**	consensus-motifs2.txt
***p*-value**	2.61e-02
***E*-value**	1.31e-01
***q*-value**	1.31e-01
**Overlap**	8
**Offset**	-2
**Orientation**	Normal

Show logo download options

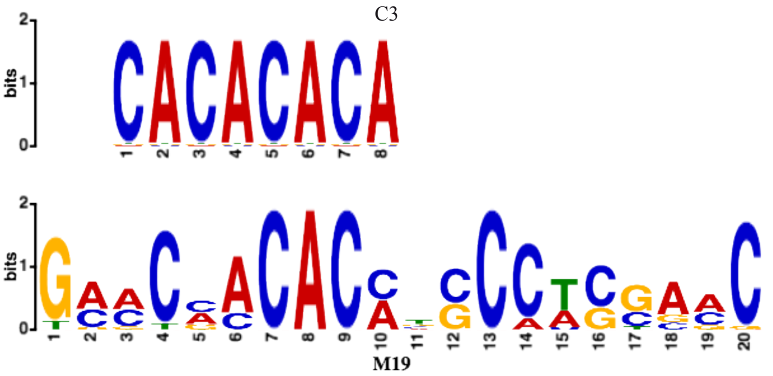

SETTINGS

Previous Next Top

Alphabet

Source: the query file

Name	Bg.				Bg.	Name
Adenine	0.25	A	~	T	0.25	Thymine
Cytosine	0.25	C	~	G	0.25	Guanine

Other Settings

**Strand Handling**	Motifs may be reverse complemented before comparison to find a better match.
**Distance Measure**	Euclidean distance
**Match Threshold**	Matches must have a q-value of 0.5 or smaller.

Previous Top

TOMTOM version
5.0.1 (Release date: Thu Jul 26 17:15:19 2018 -0700)

Reference
Shobhit Gupta, JA Stamatoyannopolous, Timothy Bailey and William Stafford Noble, "Quantifying similarity between motifs", *Genome Biology*, **8**(2):R24, 2007.

Command line

```
tomtom -oc 10TomTom --no-ssc -eps 00Motifs/adrian-motifs.txt 08ConsensusMotifs/consensus-motifs2.txt
```

Result calculation took 0.729 seconds
