# Supplementary figures and images for: DNA Motifs Are Not General Predictors of Recombination in Two Drosophila Sister Species
Source: Genome Biol Evol. 2019 Apr 15;11(4):1345–57. doi: 10.1093/gbe/evz082 (PMC6490297; doi:10.1093/gbe/evz082)

a

*D. melanogaster* at 101k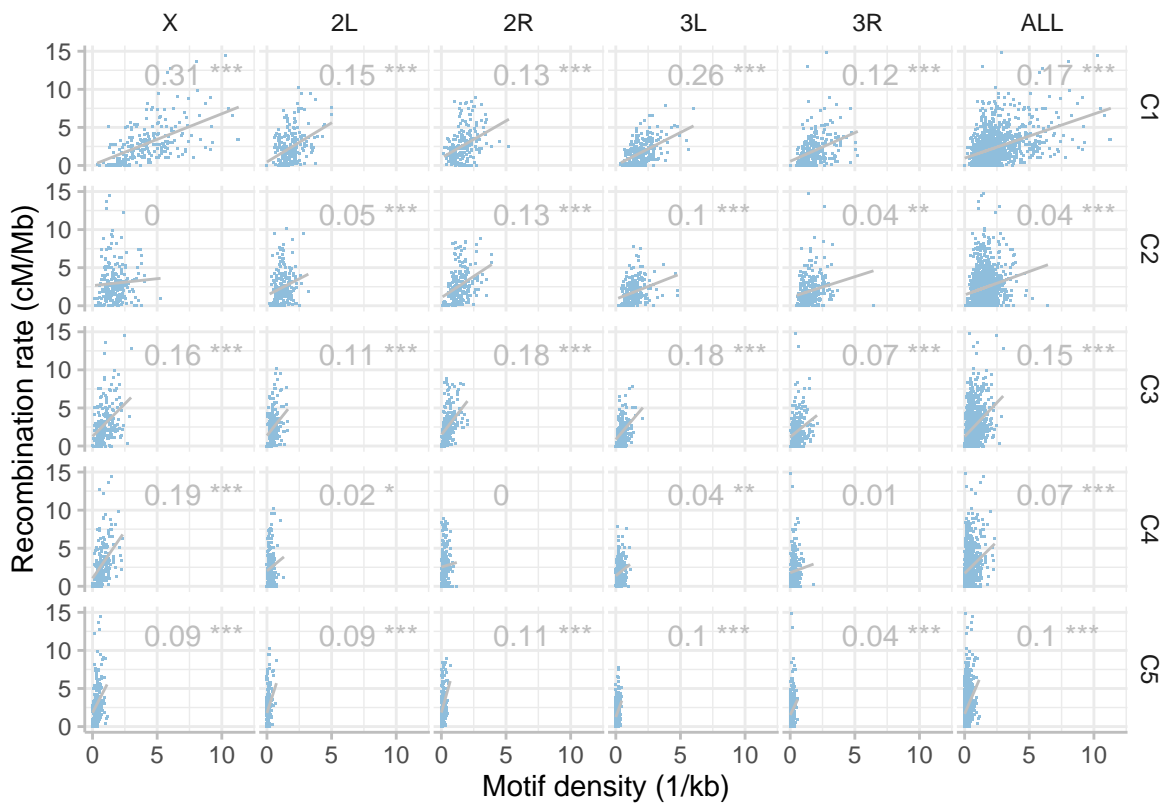

b

*D. simulans* re-binned at 101k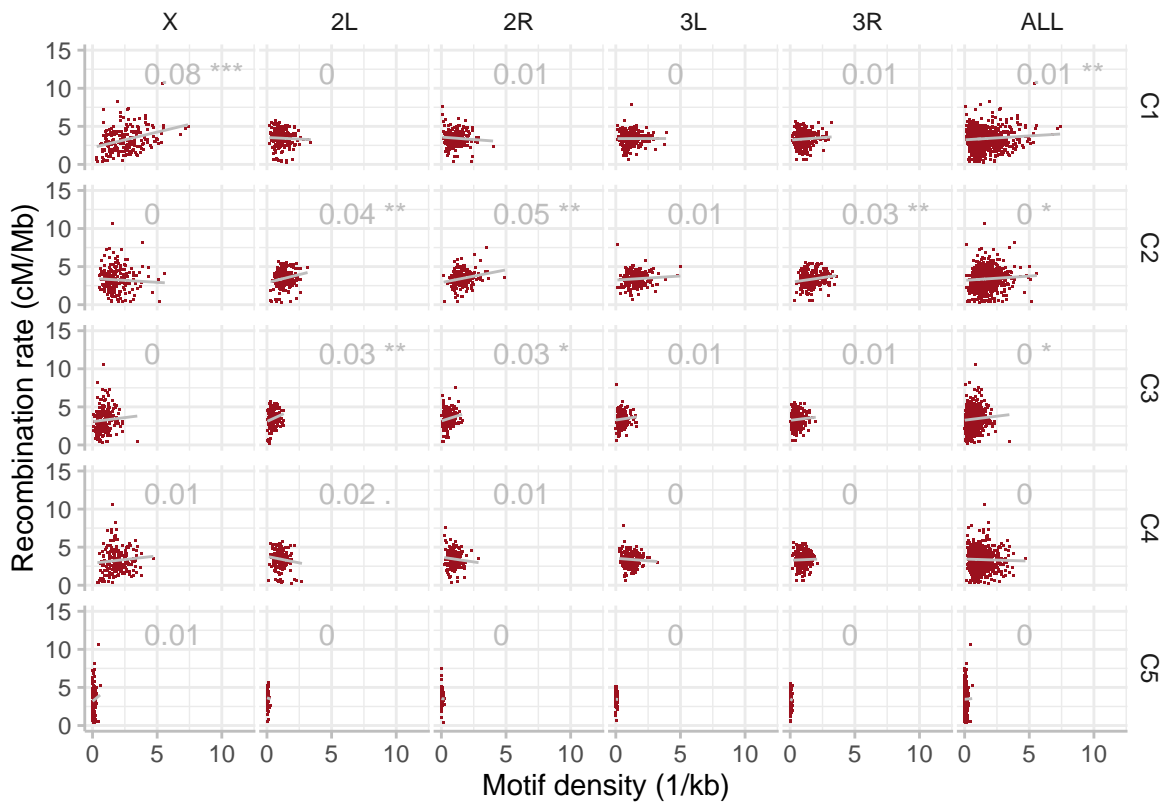

Supplement: Supplementary Data [file evz082_supp.zip › figS1-single_motif_models.pdf]

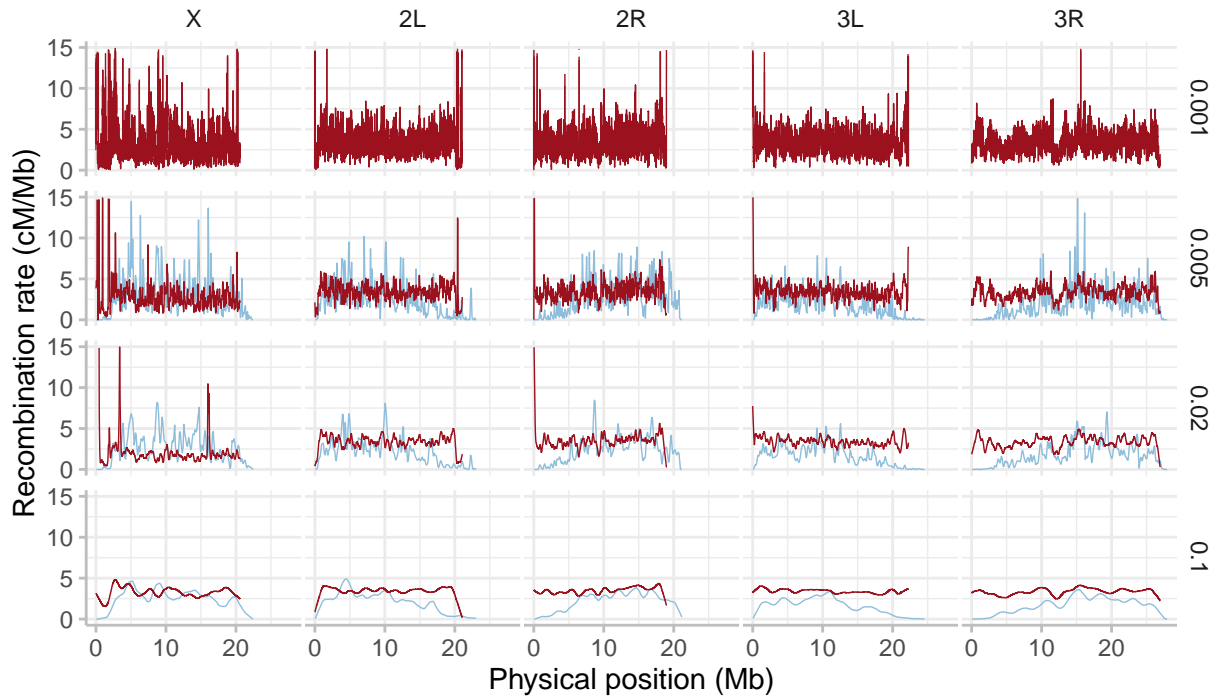

Supplement: Supplementary Data [file evz082_supp.zip › figS2-recombination-loess.pdf]
